# Nonlinear Optical Response of a Plasmonic Nanoantenna to Circularly Polarized Light: Rotation of Multipolar Charge Density and Near-Field Spin Angular Momentum Inversion

**DOI:** 10.1021/acsphotonics.3c00783

**Published:** 2023-10-24

**Authors:** Marina Quijada, Antton Babaze, Javier Aizpurua, Andrei G. Borisov

**Affiliations:** † Department of Applied Mathematics, UPV/EHU, 20018 Donostia-San Sebastián, Spain; ‡ Department of Electricity and Electronics, FCT-ZTF, UPV-EHU, 48080 Bilbao, Spain; § Materials Physics Center CSIC-UPV/EHU, Paseo Manuel de Lardizabal 5, 20018 Donostia-San Sebastián, Spain; ∥ Donostia International Physics Center (DIPC), Paseo Manuel de Lardizabal 4, 20018 Donostia-San Sebastián, Spain; ⊥ Institut des Sciences Moléculaires d’Orsay (ISMO)—UMR 8214, CNRS, Université Paris-Saclay, 91405 Orsay Cedex, France

**Keywords:** nonlinear optics, light polarization, circularly polarized light, high-harmonic generation, plasmonic nanostructure, time-dependent density functional theory

## Abstract

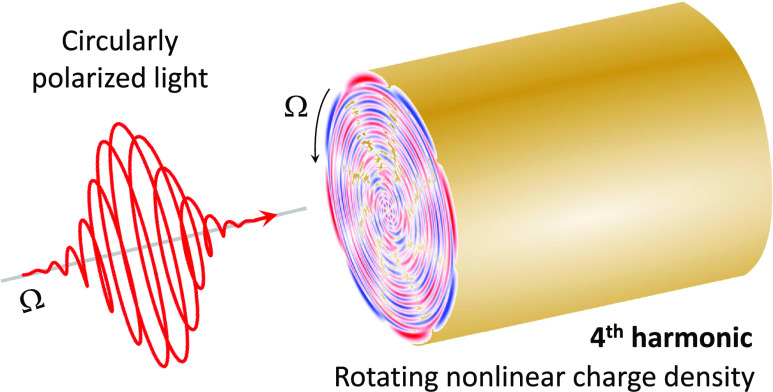

The spin and orbital angular momentum carried by electromagnetic pulses open new perspectives to control nonlinear processes in light–matter interactions, with a wealth of potential applications. In this work, we use time-dependent density functional theory (TDDFT) to study the nonlinear optical response of a free-electron plasmonic nanowire to an intense, circularly polarized electromagnetic pulse. In contrast to the well-studied case of the linear polarization, we find that the *n*th harmonic optical response to circularly polarized light is determined by the multipole moment of order *n* of the induced nonlinear charge density that rotates around the nanowire axis at the fundamental frequency. As a consequence, the frequency conversion in the far field is suppressed, whereas electric near fields at all harmonic frequencies are induced in the proximity of the nanowire surface. These near fields are circularly polarized with handedness opposite to that of the incident pulse, thus producing an inversion of the spin angular momentum. An analytical approach based on general symmetry constraints nicely explains our numerical findings and allows for generalization of the TDDFT results. This work thus offers new insights into nonlinear optical processes in nanoscale plasmonic nanostructures that allow for the manipulation of the angular momentum of light at harmonic frequencies.

## Introduction

Modern technologies enable the design and nanofabrication of photonic devices for manipulation of optical fields on spatial scales much smaller than the wavelength of light.^1^ In particular, the resonant coupling of photons with collective electronic excitations in metals and two-dimensional (2D) materials, i.e., plasmons, can be used to engineer strongly enhanced near fields confined to the atomic scale.^2−5^ Near-field enhancement boosts the nonlinear optical response so that plasmonic systems find practical applications not only in the linear^5−7^ but also in the nonlinear^8−10^ regime. Nonlinear metrology,^11^ nonlinear sensing,^12,13^ ultrafast spectroscopy,^14−16^ and nonlinear integrated photonic circuits operation^17−19^ exemplify various fields that take advantage of the nonlinear optical response of plasmonic systems.

The recent interest in the use of structured light^20^ has dynamized research on plasmonic metasurfaces exploiting spin-controlled nonlinear optical processes to obtain beam shaping through manipulation of orbital angular momentum (OAM) and spin angular momentum (SAM) of light.^21−30^ Along with gas-phase techniques,^31−34^ the use of nonlinear metasurfaces^35^ for the generation of vacuum ultraviolet (VUV) and extreme ultraviolet (XUV) coherent light that carries angular momentum opens exciting perspectives in ultrafast spectroscopies and time-resolved experiments to probe chiral systems.

The development of devices for on-chip control of nonlinear fields requires knowledge of the nonlinear optical response of individual plasmonic nanoparticles and plasmonic molecules, which are the building blocks of such nonlinear devices. To this end, the hydrodynamic description adopted to address the nonlinear response of conduction electrons, and first applied to characterize the second-harmonic generation from metals and metal surfaces,^36−41^ has been further developed recently. Efficient numerical approaches to address the nonlinearity of plasmonic nanoparticles have been thus proposed.^9,42−48^ In this context, the situation where the fundamental wave is linearly polarized has been studied both theoretically and experimentally, providing a deep understanding about the main processes that control the second-order^45−47,49−57^ and the third-order^42,58−63^ response of plasmonic nanoantennas and subnanometric plasmonic gaps prone to sustain optically assisted tunneling.^64−66^ However, with the exception of chiral systems^9,67−70^ (where one is naturally interested in the nonlinear activity triggered by SAM-carrying incident fields), the case of a circularly polarized fundamental wave interacting with typical plasmonic nanoantennas^71^ has received less attention for nonlinear plasmonic applications.

**Figure 1:**
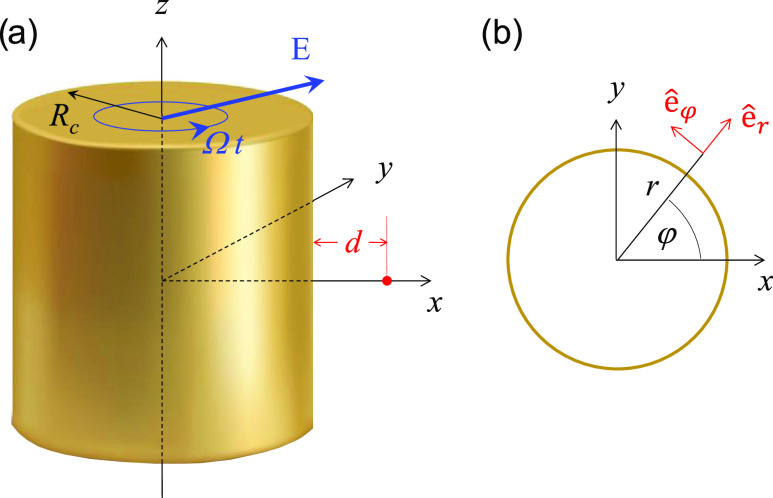
Sketch of the studied system. (a) Plasmonic nanowire of radius *R*_c_ = 66.4 *a*_0_ (≈3.5 nm) infinite along the *z*-axis. The external field is indicated with a blue arrow. It is circularly polarized in the (*x*, *y*)-plane and rotates anticlockwise (left-handed) with a fundamental frequency Ω. The red dot located at the *x*-axis at a distance *d* from the surface of the nanowire indicates the position at which we calculate the induced near field. (b) Cross-section of the nanowire in the (*x*, *y*)-plane, definition of the cylindrical coordinates used in the paper.

In this work, we address the nonlinear optical response of a plasmonic nanostructure to a SAM-carrying incident field. We use time-dependent density functional theory (TDDFT) to study the dynamics of conduction electrons triggered by an intense electromagnetic pulse in a free-electron cylindrical nanowire. The electric field of the pulse is circularly polarized in the transversal plane of the nanowire (see Figure 1). As a reference, we also perform calculations for linearly polarized fundamental field as studied in detail in previous works.^43,47,72−75^ Without any *a priori* assumptions, our TDDFT results reveal that the optical response at the *n*th harmonic of the circularly polarized fundamental wave is determined by the multipole moment of order *n* of the induced nonlinear charge density that rotates around the nanowire axis at the fundamental frequency. In particular, the induced near field is circularly polarized at all harmonics of the fundamental frequency and reveals an SAM inversion. Moreover, the frequency conversion in the far field is suppressed for circularly polarized incident pulses. We further demonstrate that these results are a direct consequence of the symmetry of the system as can be fully described and understood within an analytical approach. Therefore, our findings are qualitatively robust and provide a new paradigm for the design of nonlinear nanoscale optical devices.

Unless otherwise stated, atomic units (a.u.) are used throughout the paper.

## Methods

The details on the method including the modeling of plasmonic nanoparticles and the real-time TDDFT calculations of the electron dynamics can be found in prior works.^76,77^ Thus, only the aspects specific to this study will be described here. We consider a plasmonic nanowire represented as a free-electron metal cylinder of radius *R*_c_, infinite along the *z*-axis (see Figure 1). The nanowire is described using the stabilized jellium model^78^ characterized by the Wigner–Seitz radius of gold (and silver), *r*_s_ = 3.02 *a*_0_ (*a*_0_ = 0.0529 nm is the Bohr radius), and a work function of 5.49 eV. The radius of the nanowire is set to *R*_c_ = 66.4 *a*_0_ (≈ 3.5 nm), which is a good compromise between the feasibility of the TDDFT calculations and a sufficiently small value of the surface Landau damping^79,80^ so that well-resolved localized plasmon resonances can be observed in the linear optical response.

The free-electron model is well suited to quantitatively address the linear and nonlinear optical response of nanoparticles formed by prototype metals such as alkali metals and aluminum. For noble metals, as far as the fundamental frequency is below the onset of interband transitions involving localized d-electrons, the symmetry-protected aspects of the nonlinear optical response can be nicely understood within the framework of the free-electron model, considering that the nonlinear currents are created by the quasi-free conduction-band electrons.^9,37,38,44,47,51^ The contribution of d-electrons to the dynamical screening of the fundamental and harmonic fields can be treated in a model way;^37,38,81−84^ however, a fully quantitative assessment of linear and nonlinear properties of noble metal nanoparticles would require further studies.

The nonlinear optical response of the nanowire is triggered by a Gaussian pulse of electric field **E**(*t*) left-handed circularly polarized (SAM of 1) in the (*x*, *y*)-plane
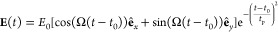
1where **ê**_*x*_ (**ê**_*y*_) stands for the unit length vector along the *x*- (*y*-) axis, *E*_0_ is the field amplitude, Ω is the fundamental frequency, *t*_p_ is the duration of the pulse, and *t*_0_ is the delay time. We also perform reference calculations for a linearly polarized fundamental field given by
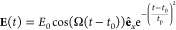
2

In this work, we use Ω = 1.5 eV,  (≈ 13.7 fs), and *E*_0_ = 0.064 × 10^–2^ – 1.07 × 10^–2^ a.u. corresponding to an average power of the circularly polarized pulse of 3.6 × 10^10^ – 1 × 10^13^ W/cm^2^, and twice smaller average power of the linearly polarized pulse. To analyze the nonlinear optical response of the nanowire to left-handed circularly polarized (SAM = 1) illumination, it is convenient to rewrite eq 1 in the form
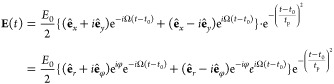
3where the second expression is given in cylindrical (*r*, φ) coordinates, and **ê**_*r*_ and **ê**_φ_ are cylindrical unit vectors (see Figure 1b).

The fundamental field given by eq 1 or eq 3 can be obtained, for instance, using *p*-polarized optical pulses where one pulse (with carrier-envelope phase equals to zero) is propagating along the *y*-axis, and another pulse (with carrier-envelope phase equals to ) is propagating along the *x*-axis. Furthermore, the present model applies to a situation in which a circularly polarized laser pulse impinges on a plasmonic nanowire along its symmetry axis. In this case, the height *h* of the nanowire has to be small compared to the wavelength so that there is no effect of plasmon propagation along the *z*-axis. Moreover, *h* has to be significantly larger than the Fermi wavelength of electrons and large enough for the top and bottom surface effects to be neglected.^76^

The dynamics of the electron density within the nanowire in response to the optical excitation is obtained from real-space, real-time TDDFT calculations within the Kohn–Sham (KS) scheme.^85,86^ Since the system is invariant with respect to a translation along the *z*-coordinate, the time-dependent electron density is sought in the form *n*(**r**, *t*) = ∑_*j*_ χ_*j*_ |ψ_*j*_(**r**, *t*)|^2^, where ψ_*j*_(**r**, *t*) are the KS orbitals of the noninteracting electron system, and **r** = (*x*, *y*) is the 2D position vector. The sum runs over the occupied KS orbitals with ground-state energies  ( is the Fermi energy), and the statistical factors  account for spin degeneracy as well as for the electron motion along the *z*-axis. The orbitals ψ_*j*_(**r**, *t*) evolve in time according to the 2D time-dependent KS equations, where the nonretarded approximation is used consistent with the small relevant dimensions of the system
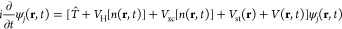
4The initial conditions ψ_*j*_(**r**, *t* = 0) correspond to the KS orbitals of the ground-state system. In eq 4, *T̂* is the kinetic-energy operator, *V*_H_[*n*(**r**, *t*)] is the Hartree potential, *V*_xc_[*n*(**r**, *t*)] is the exchange–correlation potential, and *V*_st_(**r**) is the stabilization potential. The time evolution of the electron density, *n*(**r**, *t*), introduces a time dependence to the Hartree and exchange–correlation potentials. The kernel formulated by Gunnarsson and Lundqvist^87^ within the adiabatic local-density approximation (ALDA)^86^ is used in this work to compute *V*_xc_[*n*(**r**, *t*)]. Finally, *V*(**r**, *t*) = **r**·**E**(*t*) is the potential of the optical field.

From the time evolution of the electron density, we obtain all the time-dependent quantities of interest such as the induced charge density
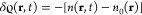
5and the induced electric near field
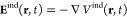
6where *n*_0_(**r**) is the electron density of the ground state of the system, and *V*^ind^(**r**, *t*) = −{*V*_H_[*n*(**r**, *t*)]– *V*_H_[*n*_0_(**r**)]} is the induced potential. We also calculate the multipole moments *Q_m_*(*t*) of the charge density induced in the nanowire per unit length in *z*
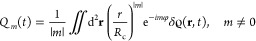
7This expression is given in cylindrical (*r*, φ) coordinates. From the charge neutrality of the system, the monopole moment is zero, *Q*_0_(*t*) = 0. The induced dipole moment per unit length can be found from . For the sake of compactness, we use the term “multipole moments” below and understand that the corresponding quantities are calculated per unit length along the *z*-coordinate.

Any frequency-resolved magnitude  reported in this work is obtained from the time-dependent result  using the time-to-frequency Fourier transform. In what follows, without loss of generality, we consider positive frequencies, ω > 0, and  as the time dependence of the spectral components. Along with the time-to-frequency Fourier transform of *Q_m_*(*t*) given by eq 7, the multipole moments *Q_m_*(ω) at harmonics ω = *n*Ω of the fundamental frequency can also be obtained from the corresponding spectral components of the induced charge density as
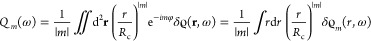
8The coefficients δϱ*_m_*(*r*, ω) are defined in the angular e^*im*φ^ basis using a representation of the induced charge density
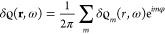
9

The multipole moments *Q_m_*(ω) given by eq 8 can be used to express the induced potential
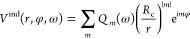
10and the near field
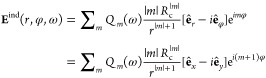
11induced at harmonic frequencies ω = *n*Ω of the fundamental field.

## Results and Discussion

### Linear Response

Prior to the discussion of the nonlinear optical response, we analyze the linear optical response and plasmon resonances of the nanowire using TDDFT. Consistent with the symmetry of the system, the plasmon modes studied here correspond to the cylinder plasmons^88−90^ in the limit of zero wavevector along the *z*-axis. These plasmon modes are localized multipolar plasmons and can be identified with the multipole order *m* associated with the e^*im*φ^ dependence of the plasmon-induced electron density, potential, and near field. The ±*m* plasmon modes are degenerate in frequency. Therefore, below we consider only positive values of *m*. The localized multipolar plasmons evolve in the (*x*, *y*)-plane and can be understood as surface plasmons sustained along the circumference of the nanowire, which leads to a quantized wavenumber .^77,91^

**Figure 2:**
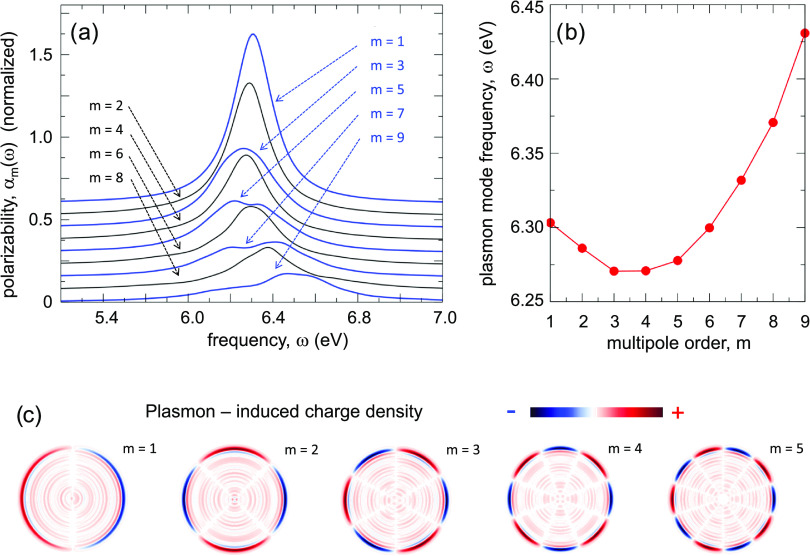
Linear optical response of a metallic nanowire calculated with TDDFT. (a) Imaginary part of the multipolar polarizabilities of order *m* per unit length, Im{α*_m_*(ω)}. Results are shown as a function of frequency. Each spectrum is vertically offset for clarity. (b) Frequency ω*_m_* of the localized multipolar plasmon resonance sustained by the metallic nanowire as a function of the multipole order *m*. (c) The induced charge density δϱ given by the coherent superposition of the ± *m* multipolar plasmon modes, for different *m* as labeled above each interpolated image. Results are shown in the (*x*, *y*)-plane, and the system is translationally invariant with respect to the *z*-axis. The red (blue) color corresponds to positive (negative) values of δϱ as indicated with a color bar.

The excitation of localized multipolar plasmons in the nanowire is revealed by a resonant profile in the frequency dependence of the multipolar polarizabilities α*_m_*(ω) calculated with TDDFT as shown in Figure 2a (see SI for the definition of α*_m_*(ω)). The analysis of the resonances yields the frequencies ω*_m_* and lifetimes τ*_m_* = 1/Γ*_m_* of the underlying plasmon modes. Here, Γ*_m_* is the full width at half maximum of the resonance. The resonant profile of Im{α*_m_*(ω)} is often perturbed by additional features related to the decay of plasmons into electron–hole pair excitations.^79,92^ We therefore define ω*_m_* not as the frequency at which Im{α*_m_*(ω)} is maximum but as its mean frequency.

The dipolar plasmon mode (*m* = 1) at ω_1_ = 6.30 eV is slightly redshifted from the classical nonretarded surface plasmon frequency ω_s_ = ω_p_/ = 6.35 eV (ω_p_ = 8.98 eV is the bulk plasma frequency) because of the spill-out of the induced electron density.^81,93,94^ Note that the plasmon frequencies of the nanowire considered here are significantly higher than those of actual noble metal nanostructures, since the dynamical screening associated with d-band electrons is not considered in our jellium model.^81,93^ However, the differences in the nonlinear response of a plasmonic system induced by a SAM-carrying fundamental field or by a linearly polarized field stem from robust symmetry properties. Thus, the influence of bound electrons on the field screening and on the plasmon resonances does not alter the qualitative findings reported here.

Interestingly, while the classical nonretarded theory predicts ω_*m*_ = ω_s_ irrespective of *m*, the frequencies of the plasmon modes calculated with TDDFT depend on *m*. As shown in Figure 2b, ω*_m_* first redshifts with increasing *m* up to *m* = 3 and then monotonically blueshifts for larger *m* ⩾ 4. This finding can be understood considering the picture of localized multipolar plasmons as surface plasmons with quantized wavenumber  propagating along the circumference of the cross-section of the nanowire in the (*x*, *y*)-plane.^77,91^ The dispersion of ω*_m_* with *m* can be then associated with the well-known dispersion relationship of the surface plasmon frequency on a planar free-electron metal surface as a function of the wavenumber *q* parallel to the surface.^82^ Finally, Figure 2c nicely illustrates the multipolar surface character of the *m* = 1–5 plasmon modes. The induced charge density is mainly located at the metal–vacuum interface and features the characteristic e^*imφ*^ angular dependence.

### Nonlinear Response

**Figure 3:**
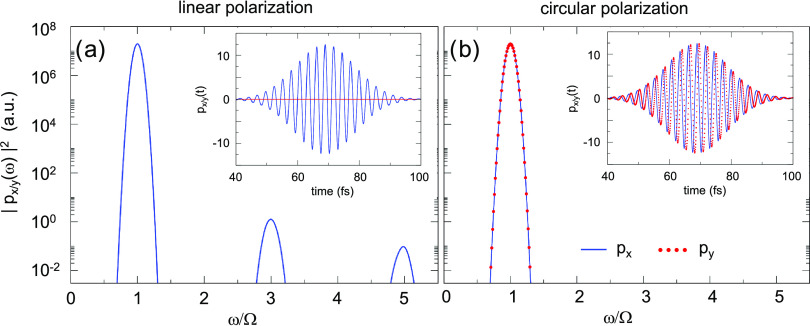
Dipolar response of the nanowire to a linearly (a) and circularly (b) polarized fundamental field with frequency Ω = 1.5 eV. The TDDFT calculations are performed for an amplitude of the fundamental field *E*_0_ = 5.2 × 10^–3^ a.u. corresponding to an average power of the circularly polarized optical pulse of 2.4 × 10^12^ W/cm^2^. The spectra |*p*_*x*_(ω)|^2^ and |*p*_*y*_(ω)|^2^ of the scalar projections of the induced dipole moment, **p**(*t*), on the *x*- (*p*_*x*_) and *y*- (*p*_*y*_) directions are shown as a function of frequency measured in units of the fundamental frequency. The blue (red dotted) line is used for *p*_*x*_ (*p*_*y*_). For the case of linear polarization, the electric field is *x*-polarized so that *p*_*y*_ = 0. All the quantities are calculated per unit length of the nanowire.

We start the discussion of the nonlinear optical response of the nanowire with the analysis of the induced dipole moment **p**, which determines the efficiency of the frequency conversion in the far field. Figure 3 summarizes our results obtained for linearly (panel a) and circularly (panel b) polarized fundamental field. The fundamental frequency Ω = 1.5 eV used here is far from the plasmon resonances of the system so that no plasmon ringing is produced in the response. The time dependence of the induced dipole moment, **p**(*t*), shown in the inset of Figure 3a,b follows the time dependence of the fundamental electric field **E**(*t*). For circularly polarized illumination, this results in a π/2 phase shift between the *p*_*x*_(*t*)- and *p*_*y*_(*t*)- components of the induced dipole, as shown in the inset of panel b. We use off-resonance incident field on purpose to avoid strong energy deposition into the nanowire that would lead to an efficient electron excitation and eventually to an electron emission with charging of the nanowire. We have explicitly checked that with the present choice of the pump pulse the calculated quantities show the expected scaling with the nonlinearity order *n* and with the amplitude of the fundamental electric field. With this off-resonance condition, however, we can only observe the resonant enhancement of the nonlinear optical response^46,53,54,60,74,75,95−99^ due to the resonance between the harmonic frequency *n*Ω and the multipolar plasmon frequency ω_*m*_ and not due to the resonance between the fundamental frequency Ω and, e.g., the dipolar plasmon frequency ω_1_ of the nanowire.

The frequency spectra of the induced dipole shown in Figure 3 reveal that the far-field emission at harmonic frequencies ω = *n*Ω (*n* > 1) is only efficient for a linearly polarized fundamental field. Consistently with the symmetry of the nanowire, the TDDFT results in Figure 3a show that a nonlinear dipole is induced at odd harmonics, allowing the emission into the far field for linearly polarized illumination.^100,101^ In contrast, for circularly polarized illumination (Figure 3b), the induced dipole is suppressed at all harmonics of the fundamental frequency so that the frequency conversion into the far field is quenched.

**Figure 4:**
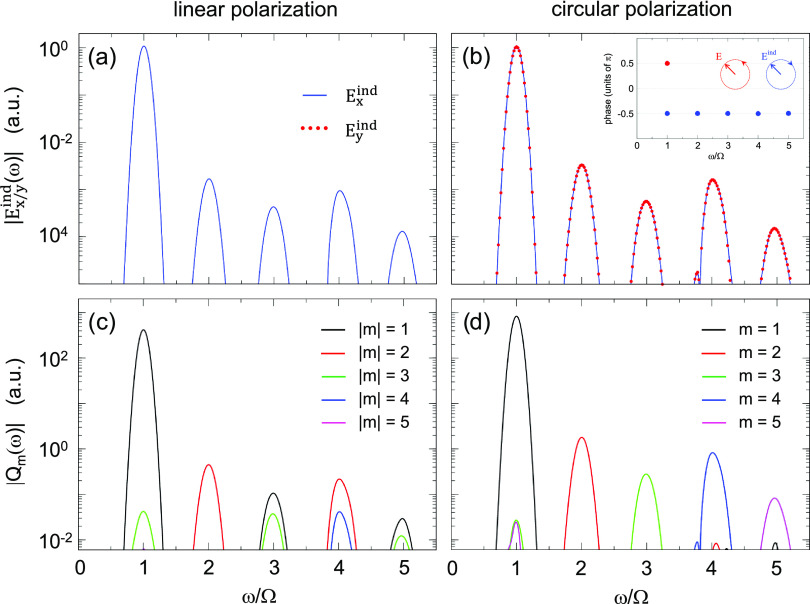
Spectral analysis of nonlinear near fields (a, b) and multipole moments (c, d) induced by the linearly (a, c) and circularly (b, d) polarized field with a fundamental frequency Ω = 1.5 eV. Results are shown as a function of the frequency measured in units of the fundamental frequency Ω. The TDDFT calculations are performed for an amplitude of the fundamental field *E*_0_ = 5.2 × 10^–3^ a.u. corresponding to an average power of the circularly polarized optical pulse of 2.4 × 10^12^ W/cm^2^. (a, b) Frequency-resolved *x*- and *y*- components of the induced near field calculated at the *x*-axis at a distance *d* = 1.3 nm from the surface of the nanowire (see geometry in Figure 1). For the color code, see the inset of panel (a). The inset of panel b shows the phase between *E*_*x*_ and *E*_*y*_ components of the near field calculated at harmonic frequencies ω = *n*Ω (blue dots). The red dot indicates the corresponding phase for the fundamental field. (c, d) Frequency-resolved multipole moments |*Q*_*m*_(ω)| of the induced charge density. The color code corresponds to the multipole order *m* as explained in the insets. For the *x*-polarized fundamental field the results obtained for ± *m* are degenerate. For a fundamental field with SAM = 1, the *Q*_*m*_(ω) are zero for nonpositive *m* ≤ 0.

The nonlinear electric near field calculated with TDDFT at a distance *d* = 1.3 nm from the surface of the nanowire is analyzed in Figure 4a,b. In contrast to the far-field emission analyzed in Figure 3, we find that all harmonics (odd and even) are present in the near field both for linear (panel a) and circular (panel b) polarizations of the fundamental field. Thus, the harmonic decomposition of the induced near field points to the presence of higher-order multipole moments (*m* > 1) of the induced nonlinear charge density. Furthermore, for left-handed circularly polarized incident field (SAM = 1) the projections of the induced nonlinear near field on *x*- and *y*- axes satisfy the relation *E*_*y*_^ind^(ω) = −*iE*_*x*_^ind^(ω) (see the – π/2 phase between *E*_*y*_^ind^(ω) and *E*_*x*_^ind^(ω) for each harmonic frequency in the inset of Figure 4b, represented with the blue dots). This relation between *x*- and *y*- field components corresponds to right-handed circular polarization (SAM = −1) of the induced nonlinear near field, which is opposite to that of the fundamental field. The inversion of the SAM between the fundamental field and the nonlinear near field holds for all harmonic frequencies encompassed here. We demonstrate below with an analytical approach that this SAM inversion stems from the symmetry of the system and that it is not specific to a given observation point but rather a general property of the near field.

The excitation of nonlinear multipole moments at harmonic frequencies *n*Ω is evidenced with the results shown in Figure 4c,d. In this figure, we show the spectral analysis of the time-dependent multipole moments *Q*_*m*_(*t*) of the induced charge density δϱ(**r**, *t*) calculated with TDDFT using eq 7. The multipole moments *Q*_*m*_(ω), obtained from the time-to-frequency Fourier transform of *Q*_*m*_(*t*), feature well-resolved harmonic contributions at ω = *n*Ω. At a fixed harmonic frequency *n*Ω, for a linearly polarized incident field (Figure 4c) one or several multipole moments *Q*_*m*_(*n*Ω) of the nonlinear induced charge density are excited with ±*m* degeneracy, and |*m*| = *n* – 2*j* (*j* = 0, 1..., and 2*j* < *n*). In particular, multipole moments *Q*_±1_(ω) of order *m* = ± 1 (and thus a dipole moment **p**(ω) given by their linear combination) are present at odd harmonics, and absent at even harmonics, consistent with the results reported in Figure 3a. For a circularly polarized incident field (Figure 4d), a qualitatively different nonlinear response is obtained. Namely, only multipole moments *Q*_*n*_(*n*Ω) with positive *m* = *n* are excited in this situation, i.e., the nonlinear induced charge density is characterized by the multipole moment of the same order *m* as the harmonic order *n*.

It is also worth noting that the fourth harmonic of the fundamental frequency Ω = 1.5 eV overlaps the localized multipolar plasmon *m* = 4 of the nanowire. This occurs because of the finite width, Γ_*m*_, of the latter (see Figure 2a,b) allowing for the condition  to be fulfilled. The *m*-order multipolar plasmon is characterized by the e^*im*φ^ angular dependence of the surface charges (see Figure 2c). The component of the induced nonlinear charge density δϱ_*m*_(*r*,ω) e^*im*φ^ (see eq 9) which contributes to the multipole moment *Q*_*m*_ (see eq 8) has the same angular dependence. This leads to a resonant enhancement of the multipole moment *Q*_4_(4Ω) for the circularly polarized fundamental field. Indeed, the amplitude of *Q*_4_(4Ω) stands off the general trend in Figure 4d that shows a decreasing sequence of |*Q*_*n*_(*n*Ω)| with increasing *n*. The resonance enhancement is also observed in Figure 4c for the multipole moments *Q*_±2_(4Ω) and *Q*_±4_(4Ω) excited by linearly polarized fundamental field (see also ref (101) for the discussion of the resonant enhancement of the fourth-harmonic generation in a polarized spherical Al nanoparticle).

### Analytical Interpretation of the TDDFT Results

The main physics behind the TDDFT results can be understood using an approach^102,103^ often evoked in the context of nonlinear metamaterials,^22−24,35^ which is based on the symmetry of the system and Neumann’s principle for tensors.^104,105^ To this end, it is convenient to use cylindrical coordinates and to introduce the basis of the anticlockwise (SAM = +1) and clockwise (SAM = −1) rotating waves defined as
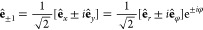
12The fundamental field **E**(Ω) can be expressed in the **ê**_±1_ basis as follows:
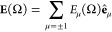
13which is position-independent in the absence of retardation effects. For circularly polarized illumination, eq 13 results in **E**(Ω) = *E*_±1_(Ω)**ê**_±1_, where ±1 stands for the SAM of the fundamental field. For linearly polarized illumination **E**(Ω) = *E*_+1_(Ω)**ê**_+1_ + *E*_–1_(Ω)**ê**_–1_, where *E*_–1_(Ω) = *E*_+1_(Ω) for an *x*-polarized incident field and *E*_–1_(Ω) = −*E*_+1_(Ω) for a *y*-polarized incident field. For circular and linear polarizations, **E**(−Ω) = [**E**(Ω)]*, where [*Z*]* stands for the complex conjugate of a complex number *Z*.

Using the basis of rotating waves, we introduce the nonlinear multipolar hyperpolarizabilities α_*m*;μ_1_...μ_*n*__^(*n*)^ as
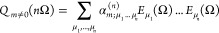
14where *Q*_*m*_(ω = *n*Ω) is the *m*th order multipolar moment of the charge density induced in the nanowire per unit length (eq 8), and indexes μ_1_,...,μ*_n_* can take values of ±1 independently. Here, we consider the smallest possible nonlinear order in the field and neglect the contribution of the higher-order terms such as ∝*E*_μ_1__(Ω)...*E*_μ_*n*__(Ω)*E*_μ_*n*+1__(Ω)*E*_μ_*n*+2__(−Ω). Let us perform an anticlockwise rotation of the *x*- and *y*- axes by an angle of β around the nanowire axis. In cylindrical coordinates, this rotation is expressed as φ = φ′ + β, where the variable with an apostrophe refers to the rotated coordinate system. Consequently, the vector components are transformed as *E*_μ_^′^(ω) = e^+*i*μβ^*E*_μ_(ω), the multipolar moments are transformed as *Q*_*m*_^′^(ω) = e^+*im*β^*Q*_*m*_(ω), and the multipolar hyperpolarizabilities are transformed as
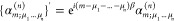
15

For a nanowire with axial symmetry, the Neumann’s principle^104,105^ implies that a rotation by any angle must preserve the form of the multipolar hyperpolarizability tensor. The following selection rule is then obtained
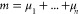
16The hyperpolarizabilities α_*m*;μ_1_...μ_*n*__^(*n*)^ are nonzero only if eq 16 is fulfilled.

Consider first a linearly polarized fundamental field, which contains both components *E*_±1_(Ω) equal in absolute value in the basis of **ê**_±1_. The condition given by eq 16 can be fulfilled by several combinations of (*m*; μ_1_, ..., μ_*n*_) with *m* = ± |*n* – 2*j*| (*j* = 0, 1, ..., and 2*j* < *n*). Consequently, only the corresponding *Q*_*m*_(*n*Ω) multipole moments are excited. For example, for the second harmonic *n* = 2, we have (±2; ± 1, ± 1) leading to the formation of a quadrupole moment. As another example, for the third harmonic *n* = 3, we have (±3; ± 1, ± 1, ± 1), leading to an octupole moment, and (±1; ± 1, ± 1, ∓1), leading to a dipole moment. Notice that the nonlinear dipole moment (*m* = ±1), and thus the frequency conversion into the far field, is possible only for odd harmonics consistent with the symmetry of the system, as shown in the TDDFT results in Figure 3a.

Consider now a left-handed circularly polarized fundamental field (SAM = 1). The only nonvanishing component in the **ê**_±1_ basis is *E*_+1_(ω). Therefore, μ_1_ = μ_2_ = ... = μ_*n*_ = 1, and μ_1_ + ... + μ_*n*_ = *n*. It thus follows from eq 16 that, in this situation, the nonlinear response at the *n*th harmonic of the fundamental frequency exclusively allows for the formation of an *n*-order multipole moment *Q*_*n*_(*n*Ω) of the induced charge density δϱ(**r**, *n*Ω). For *n* ≥ 2, the nonlinear dipole moment is zero so that the frequency conversion into the far field is suppressed, as shown in the TDDFT results in Figure 3b. The induced nonlinear near field can be obtained from eq 11, and it is given by . That is, for the circularly polarized fundamental field with SAM = +1, the induced field is circularly polarized with SAM = −1. The SAM inversion is obtained in the near field irrespective of the position and for all harmonics.

The general consequences of the symmetry of the system deduced above explain the TDDFT results reported in Figure 3 and in Figure 4 when changing from linear polarization of the fundamental field to circular polarization, namely: (i) suppression of the frequency conversion into the far field, (ii) SAM inversion in the nonlinear near field for all harmonics of the fundamental frequency, and (iii) exclusive formation of Q_*n*_(*n*Ω) multipole moment of the nonlinear charge density at *n*Ω harmonic frequency for circularly polarized illumination.

### Rotating Nonlinear Charge Density

**Figure 5:**
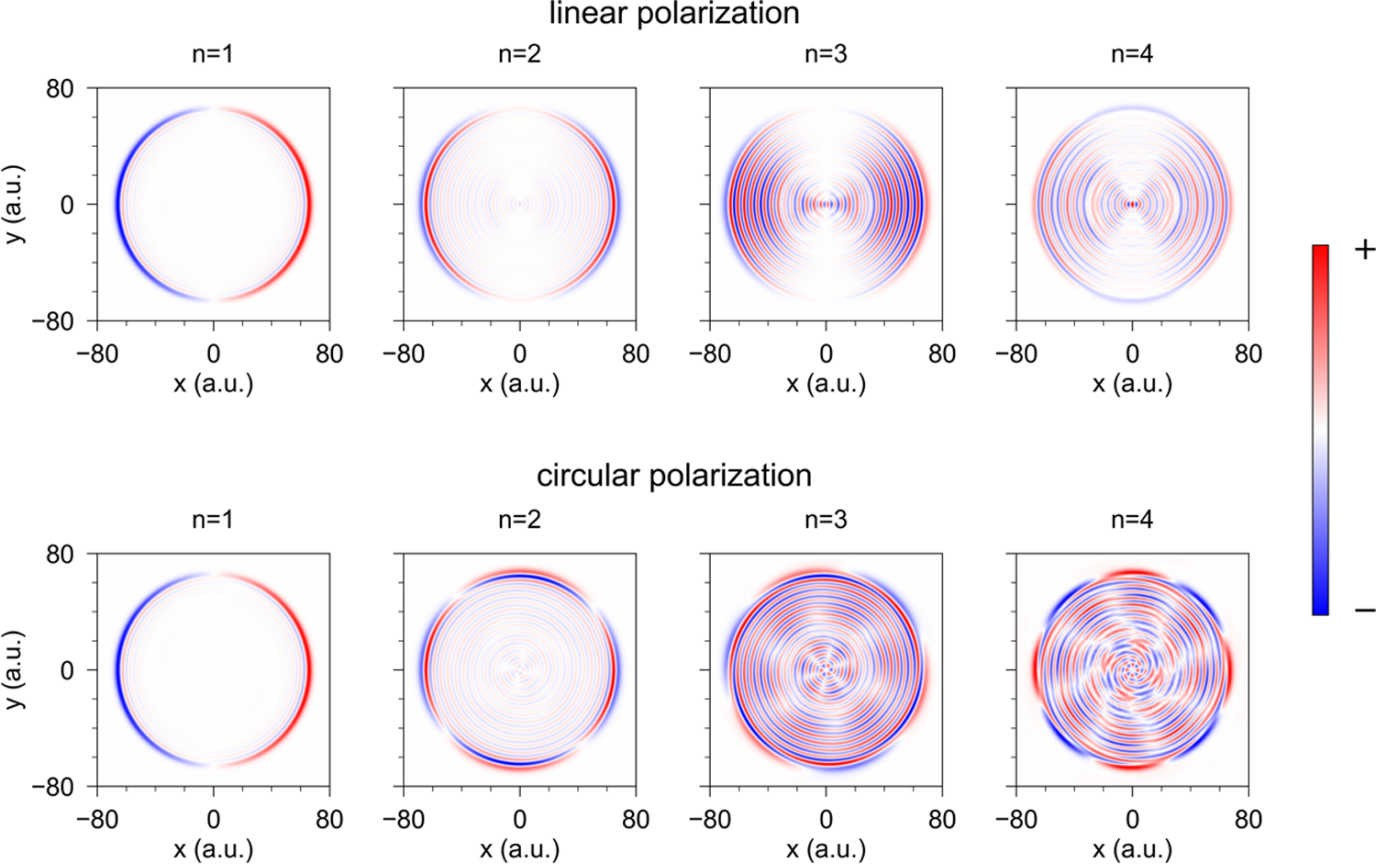
Maps of the nonlinear charge density Re{δϱ(**r**, *n*Ω)}, *n* = 1, 2, 3, 4, induced in a cylindrical nanowire at a fundamental frequency Ω = 1.5 eV, and at higher harmonics. Re{*Z*} stands for the real part of the complex number *Z*. The nonlinear charge density is shown as a function of *x*- and *y*-coordinates in the transverse plane of the nanowire. Results are normalized independently for each panel such that the variation is contained within the [−1, + 1] interval. The color scale is defined with color bars at the right of the figure. The upper (lower) row of panels show results obtained with linear (circular) polarization of the fundamental field. The TDDFT calculations were performed for an amplitude of the fundamental field *E*_0_ = 1.1 × 10^–2^ a.u. corresponding to an average power of the circularly polarized pulse 10^13^ W/cm^2^.

To gain deeper insight into the nonlinear response of the system and to validate the theoretical analysis presented above, we show in Figure 5 the maps of the charge density Re{δϱ(**r**, *n*Ω)} induced at the fundamental frequency Ω (*n* = 1; linear response), and at higher harmonic frequencies *n*Ω (*n* = 2, 3, 4; nonlinear response). Re{*Z*} stands for the real part of the complex number *Z*. The TDDFT results are shown as a function of *x*- and *y*-coordinates in the transversal plane of the nanowire for linear (upper row of panels) and circular (lower row of panels) polarizations of the fundamental field. The dipolar character of the induced density characterizing the linear response (*n* = 1) can be clearly seen for both polarizations. Obviously, for circularly polarized fundamental field, the induced dipole must rotate following the direction given by the electric field vector.

In the absence of retardation effects, for linearly polarized illumination, the nonlinear response of the free-electron homogeneous nanowire should be driven by the surface polarization at even harmonics, and predominantly by the bulk polarization at odd harmonics^9,38,39,44,46,51^ (see discussion in the SI). We indeed observe this trend for the second (*n* = 2) and third (*n* = 3) harmonics. However, for the fourth harmonic (*n* = 4), the bulk contribution to δϱ(**r**, 4Ω) is also clearly visible in addition to the nonlinear charges generated at the surface. Similarly, for circularly polarized illumination, the nonlinear polarization of a homogeneous nanowire is possible only at the surface owing to the symmetry break (see the discussion in the SI). This is consistent with the results obtained for *n* = 2. However, for higher harmonics, δϱ(**r**, *n*Ω) has a strong bulk component. We attribute this effect to the break of the homogeneous approximation because of the Friedel oscillations of the ground-state charge density. As we show in the SI, Friedel oscillations persist in the bulk of the nanowire because of its relatively small radius (see also discussion of the currents induced inside a plasmonic nanoparticle in ref (71)).

The most important information stems, however, from the symmetry of the charge density maps. For linearly polarized fundamental field (upper panels in Figure 5), Re{δϱ(**r**, *n*Ω)} is symmetric with respect to the *x*-axis, and from the symmetry with respect to the *y*-axis it follows that the dipole moment is formed only at odd harmonics. In sheer contrast, for circularly polarized fundamental field (lower panels in Figure 5), the induced charge density Re{δϱ(**r**, *n*Ω)} at harmonic frequency ω = *n*Ω possesses a well-defined axial symmetry of order *n* with respect to the nanowire axis *z*. Using the many-body response theory, we show in the SI that because of the axial symmetry of the nanowire, only the term with *m* = *n* is nonzero in eq 9. The nonlinear charge density induced at harmonic frequency can be thus expressed as  so that only *Q*_*n*_(*n*Ω) multipolar moment can be excited in full agreement with the conclusions derived from Neumann’s principle. It is however important to realize that the inverse is not true, i.e., the selection rule *Q*_*m*≠*n*_(*n*Ω) = 0 can also be satisfied if ∫*r*^|*m*|+1^d*r*δϱ_*m*_(*r*, *n*Ω) = 0 (see eq 8) and does not necessarily require that δϱ_*m*≠*n*_(*r*, *n*Ω) = 0.

The e^*inφ*^ angular dependence of the nonlinear charge density at harmonic *n* of the fundamental frequency obtained for circularly polarized illumination has appealing consequences for the dynamics of the system. Indeed, in this situation, the time evolution of the nonlinear charge density of harmonic *n* is given by
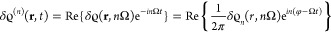
17Regardless of the harmonic order *n*, the time evolution of the nonlinear induced charge density δϱ^(*n*)^(**r**, *t*) can be seen as a rotation around the *z*-axis of the rigid multipolar charge distribution calculated for *t* = 0, . The angular frequency of this rotation equals the fundamental frequency Ω, and the direction of the rotation is the same as that of the circularly polarized fundamental field. Consequently, the oscillation frequency of the nonlinear multipole moments, near fields, and other physical quantities at the *n*th harmonic frequency stem from the symmetry of this rotating charge distribution, where the same spatial profile is retrieved in a fixed reference frame *n* times per fundamental period.

For linearly *x*-polarized fundamental field, the induced nonlinear charge density can be expressed as
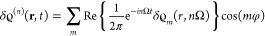
18where *m* = *n* – 2*j* (*j* = 0, 1, ..., and 2*j* < *n*). Equation 18 thus reflects the superposition of several charge distributions with angular dependencies cos(*m*φ), each of them oscillating at harmonic frequency *n*Ω. The movies presented in the SI nicely demonstrate the striking difference in the dynamics of the nonlinear charges induced at harmonic frequencies by circularly (SAM = 1) and linearly polarized fundamental field.

**Figure 6:**
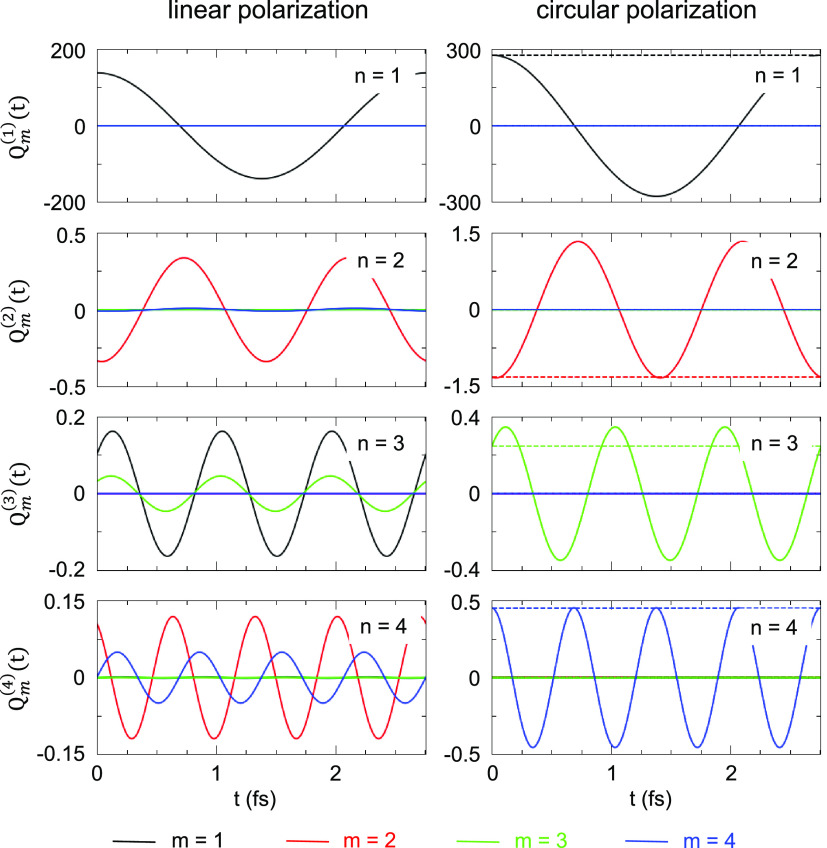
Time evolution of the multipolar moments *Q*_*m*_^(*n*)^(*t*) for linearly and circularly polarized incident fields (left and right column of panels, respectively). Here, *m* stands for the order of the multipole, and *n* stands for the harmonic order. The *Q*_*m*_^(*n*)^(*t*) are obtained using the frequency-resolved maps of the induced charge density δϱ(**r**, *n*Ω) (see Figure 5). Results are shown as a function of time for the linear response (*n* = 1) as well as for *n* = 2, 3, 4 harmonics of the fundamental frequency Ω = 1.5 eV. In the case of circular polarization, we indicate with dashed lines the time dependence of the corresponding multipolar moments calculated in a frame rotating around the *z*-axis anticlockwise with angular frequency Ω. For further details, see the text of the paper.

To further test the validity and consistency of our analysis, we use δϱ(**r**, *n*Ω) obtained from TDDFT to calculate the multipolar moments *Q*_*m*_(*n*Ω) using eq 8. The time evolution of the multipole moments at harmonic frequencies *n*Ω of a monochromatic fundamental field is given by *Q*_*m*_^(*n*)^(*t*) ≡ Re{*Q*_*m*_(*n*Ω)e^–*in*Ω*t*^}, and it is shown in Figure 6. These results are in full agreement with the results of the Fourier analysis of the time-dependent quantities defined by eq 7 and calculated using a Gaussian envelope of the fundamental field (Figure 4c,d). Thus, while several multipole moments are present at harmonic frequency *n*Ω for linearly polarized illumination (left panels), for circularly polarized illumination (right panels), the only nonzero multipole moment of the nonlinear charge density at *n*th harmonic is *Q*_*n*_^(*n*)^(*t*). When the multipolar moments are calculated not in a fixed reference frame but in a rotating frame designed to accompany the nonlinear induced charges, their time dependence is entirely removed for the circularly polarized fundamental field (dashed lines in the lower row of panels in Figure 6). This finding confirms our conclusion on the dynamics of the induced nonlinear charge density given by the anticlockwise rotation of the rigid multipolar density distribution around the symmetry *z*-axis with the angular frequency Ω.

## Summary and Conclusions

In summary, we used TDDFT calculations to study the nonlinear optical response of a free-electron plasmonic nanowire a few nanometers in diameter to a strong optical field circularly and linearly polarized within the plane perpendicular to the nanowire axis. We addressed the dependence of the nonlinear response on the polarization of the fundamental field and the possibility of generating SAM-carrying harmonics of the fundamental frequency. An analytical approach based on the symmetry of this homogeneous system and Neumann’s principle for the tensors^103−105^ was used to elucidate the main physics behind the TDDFT results obtained without any aprioristic assumptions.

In full agreement, the analytical approach and the TDDFT simulations reveal that, in this system, the nonlinear optical response to a circularly polarized fundamental field at the *n*th harmonic of the fundamental frequency Ω is driven by a nonlinear induced charge density with discrete *n*-fold symmetry with respect to the nanowire axis. This charge density rotates at the fundamental frequency Ω around the nanowire axis, and its only nonzero multipole moment is the *n*-order multipole *Q*_*n*_(*n*Ω). As a consequence, for a circularly polarized fundamental field:

- The induced density, induced field, and induced potential at *n*th harmonic frequency display a cos[*n*(φ – Ω*t*) + ζ] dependence on time and azimuthal angle φ in cylindrical coordinates, with *z*-axis along the nanowire axis (ζ is some constant).
- The frequency conversion into the far field is forbidden for all harmonics, in contrast to the case of linearly polarized illumination where the symmetry constraints permit the frequency conversion into the far field at odd harmonics.
- All harmonic frequencies are present in the near field.
- Regardless of the position, the nonlinear near field at harmonic frequencies is circularly polarized in the plane transversal to the nanowire axis, with SAM opposite to that of the fundamental field. In other words, we obtain a SAM inversion at all harmonic frequencies.

One of the main take-home messages of the present work is the striking difference in the origin of the time dependence of the physical quantities at the harmonics of the fundamental frequency, depending on the polarization of the fundamental field. We demonstrated that, in the case of circular polarization, the time dependence at the *n*th harmonic frequency stems from the order *n* multipolar symmetry of the nonlinear charge density, which rotates with angular frequency Ω (fundamental frequency) around the nanowire axis. The same spatial distribution of these charges is then retrieved in a fixed reference frame, *n*-times per fundamental period. In the case of linear polarization, the time dependence comes from several interfering charge distributions oscillating at the harmonic frequency *n*Ω.

Obviously, the quantitative results reported here depend on the specific characteristics of the free-electron plasmonic nanowire used in our work. Nevertheless, it is important to note that the qualitative conclusions obtained are derived from the symmetry of the system, and thus, they can be applied to a variety of canonical plasmonic nanoantennas. For a circularly polarized fundamental field, the SAM inversion in the near field should be observed for systems with cylindrical geometry, while the axial symmetry of the plasmonic nanoobject is the only requirement for the formation of a rotating nonlinear charge distribution with a multipolar symmetry order equal to the order of the frequency harmonic.

This work gets along the lines of active research devoted to the manipulation and control of light pulses carrying spin and angular momentum. In particular, our results pave the way toward the use of nanosources of circularly polarized high-harmonic near fields for on-chip nonlinear applications. Furthermore, our work contributes to the development of nonlinear metasurfaces for the manipulation of the angular momentum of light, since individual plasmonic nanoantennas can serve as meta-atoms to build such metasurfaces.

## Supporting Information Available

Expression for the circularly polarized fundamental field convenient for an analysis of nonlinear effects; definition of the charge multipoles in cylindrical coordinates and induced potential of the nanowire; analysis of the induced nonlinear near field; definition of the linear multipolar polarizabilities; discussion on the time-to-frequency Fourier transform used to analyze the time-dependent results of the TDDFT and to obtain the frequency-resolved quantities; classical nonretarded calculations of the linear response; extended discussion of the symmetry constraints for the bulk contribution to nonlinear polarization; derivation of the selection rules based on the nonlinear density response formalism; results showing magnetization of the nanowire by a circularly polarized fundamental field pulse; and discussion of the Friedel oscillations of the ground-state electron density (PDF)
Time evolution of the charge density induced in the nanowire, δϱ^(*n*)^(**r**, *t*) = Re{δϱ(**r**, *n*Ω)e^–*in*Ω*t*^ }, at the fundamental frequency (*n* = 1) for circular polarization with SAM = 1 of the incoming field (MP4)
Time evolution of the charge density induced in the nanowire, δϱ^(*n*)^(**r**, *t*) = Re{δϱ(**r**, *n*Ω)e^–*in*Ω*t*^ }, at the *n* = 2 harmonic for circular polarization with SAM = 1 of the incoming field (MP4)
Time evolution of the charge density induced in the nanowire, δϱ^(*n*)^(**r**, *t*) = Re{δϱ(**r**, *n*Ω)e^–*in*Ω*t*^ }, at the *n* = 3 harmonic for circular polarization with SAM = 1 of the incoming field (MP4)
Time evolution of the charge density induced in the nanowire, δϱ^(*n*)^(**r**, *t*) = Re{δϱ(**r**, *n*Ω)e^–*in*Ω*t*^ }, at *n* = 4 harmonic for circular polarization with SAM = 1 of the incoming field (MP4)
Time evolution of the charge density induced in the nanowire, δϱ^(*n*)^(**r**, *t*) = Re{δϱ(**r**, *n*Ω)e^–*in*Ω*t*^ }, at the fundamental frequency (*n* = 1) for linear polarization of the incoming field (MP4)
Time evolution of the charge density induced in the nanowire, δϱ^(*n*)^(**r**, *t*) = Re{δϱ(**r**, *n*Ω)e^–inΩ*t*^ }, at the *n* = 2 harmonic for linear polarization of the incoming field (MP4)
Time evolution of the charge density induced in the nanowire, δϱ^(*n*)^(**r**, *t*) = Re{δϱ(**r**, *n*Ω)e^–*in*Ω*t*^ }, at the *n* = 3 harmonic for linear polarization of the incoming field (MP4)
Time evolution of the charge density induced in the nanowire, δϱ^(*n*)^(**r**, *t*) = Re{δϱ(**r**, *n*Ω)e^–inΩ*t*^ }, at the *n* = 4 harmonic for linear polarization of the incoming field (MP4)
The dataset corresponding to the results shown in the figures of this paper can be found at: http://hdl.handle.net/10261/337623.
