# Supplementary material for: Nonlinear Optical Response of a Plasmonic Nanoantenna to Circularly Polarized Light: Rotation of Multipolar Charge Density and Near-Field Spin Angular Momentum Inversion
Source: ACS Photonics. 2023 Oct 24;10(11):3963–75. doi: 10.1021/acsphotonics.3c00783 (PMC10659104; doi:10.1021/acsphotonics.3c00783)
Supplement: Supplementary file 1 — ph3c00783_si_001.pdf [file ph3c00783_si_001.pdf]

Supporting Information for: Nonlinear Optical Response of a Plasmonic Nanoantenna to Circulary Polarized Light: Rotation of Multipolar Charge Density and Near-Field Spin Angular Momentum Inversion

Marina Quijada,† Antton Babaze,‡,¶,§ Javier Aizpurua,*,¶,§ and Andrei G. Borisov*,‖

†*Department of Applied Mathematics, UPV/EHU, 20018 Donostia-San Sebastián, Spain*

‡*Department of Electricity and Electronics, FCT-ZTF, UPV-EHU, 48080 Bilbao, Spain*

¶*Materials Physics Center CSIC-UPV/EHU, Paseo Manuel de Lardizabal 5 20018, Donostia-San Sebastián, Spain*

§*Donostia International Physics Center (DIPC), Paseo Manuel de Lardizabal 4 20018, Donostia-San Sebastián, Spain*

‖*Institut des Sciences Moléculaires d'Orsay (ISMO) - UMR 8214, CNRS, Université Paris-Saclay, 91405 Orsay Cedex, France*

E-mail: aizpurua@ehu.eus; andrei.borissov@universite-paris-saclay.fr

Contents

S1 Circularly polarized fundamental field. Cartesian and cylindrical coordinates. S3

S2 Charge multipoles in cylindrical coordinates and induced potential of the nanowire S4

S3 Induced nonlinear near field S5

S4 Linear multipolar polarizabilities S6

S5 Time-to-frequency Fourier transform of nonlinear quantities S8

S6 Electric field inside a cylindrical nanowire S9

S7 Symmetry-based analysis of the bulk contribution to nonlinear polarization S12

S8 Derivation of the selection rules based on the nonlinear density response formalism S14

S9 Optical magnetism S17

S10 Friedel oscillations of the electron density S20

S11 Description of movies S21

References S21

S1 Circularly polarized fundamental field. Cartesian and cylindrical coordinates.

For continuous plane-wave illumination, a fundamental electric field circularly polarized in the (x, y)- plane (see Figure 1 of the main text) is given by

$$\mathbf{E}(t) = E_0 \left[\hat{\mathbf{e}}_x \cos \Omega t \pm \hat{\mathbf{e}}_y \sin \Omega t\right], \tag{S1}$$

where E_0 is the field amplitude, "plus" sign stands for an anti-clockwise rotating field (spin angular momentum SAM= $+1$), and "minus" sign stands for a clockwise rotating field (spin angular momentum SAM= -1). Eq. S1 can be written in the form

$$\begin{aligned}\mathbf{E}(t) &= \frac{E_0}{2}\left[\hat{\mathbf{e}}_x\left(e^{-i\Omega t} + e^{i\Omega t}\right) \pm i\hat{\mathbf{e}}_y\left(e^{-i\Omega t} - e^{i\Omega t}\right)\right] \\ &= \frac{E_0}{2}\left[\hat{\mathbf{e}}_x \pm i\hat{\mathbf{e}}_y\right] e^{-i\Omega t} + \frac{E_0}{2}\left[\hat{\mathbf{e}}_x \mp i\hat{\mathbf{e}}_y\right] e^{i\Omega t}.\end{aligned} \tag{S2}$$

We consider an $e^{-i\omega t}$ time-dependence of the harmonic signals so that

$$\mathbf{E}_{\mathrm{SAM}=\pm 1}(\Omega) = \frac{E_0}{2}\left[\hat{\mathbf{e}}_x \pm i\hat{\mathbf{e}}_y\right], \tag{S3}$$

and $\mathbf{E}_{\mathrm{SAM}=\pm 1}(-\Omega) = \mathbf{E}^*_{\mathrm{SAM}=\pm 1}(\Omega)$, where * denotes the complex conjugate.

From the relations between the coordinate vectors in cartesian $(\hat{\mathbf{e}}_x, \hat{\mathbf{e}}_y)$ and cylindrical $(\hat{\mathbf{e}}_r, \hat{\mathbf{e}}_\varphi)$ coordinates

$$\begin{aligned}\hat{\mathbf{e}}_x &= \cos(\varphi)\hat{\mathbf{e}}_r - \sin(\varphi)\hat{\mathbf{e}}_\varphi, \\ \hat{\mathbf{e}}_y &= \sin(\varphi)\hat{\mathbf{e}}_r + \cos(\varphi)\hat{\mathbf{e}}_\varphi,\end{aligned} \tag{S4}$$

we obtain

$$\mathbf{E}_{\mathrm{SAM}=\pm 1}(\Omega) = \frac{E_0}{2}\left[\hat{\mathbf{e}}_r \pm i\hat{\mathbf{e}}_\varphi\right] e^{\pm i\varphi}. \tag{S5}$$

S2 Charge multipoles in cylindrical coordinates and induced potential of the nanowire

In this section, we calculate the frequency-resolved induced potential of a cylindrical nanowire interacting with a time-dependent external field, and we introduce the concept of (nonlinear) multipolar moments. The non-retarded approximation is used consistently with the small diameter of the nanowire, so that all magnitudes such as the induced charge density, potential and field depend only upon the 2D position vector $\mathbf{r}$ given by the spatial variables (r, φ) (see Figure 1 of the main text).

The induced potential $V^{\mathrm{ind}}(\mathbf{r}, \omega)$ can be sought in the form

$$V^{\mathrm{ind}}(r, \varphi, \omega) = \frac{1}{2\pi} \sum_{m} \phi_m(r, \omega)\; e^{im\varphi}, \tag{S6}$$

where the sum formally extends from $m = -\infty$ to $m = \infty$. Using Eq. (S6), the induced electric field can be obtained from $\mathbf{E}^{\mathrm{ind}}(\mathbf{r}, \omega) = -\nabla V^{\mathrm{ind}}(\mathbf{r}, \omega)$.

The induced potential is given by the solution of Poisson's equation, $\nabla^2 V^{\mathrm{ind}}(\mathbf{r}, \omega) = -4\pi \delta\varrho(\mathbf{r}, \omega)$, where $\delta\varrho(\mathbf{r}, \omega)$ is the induced charge density. Thus, the radial part of the induced potential, $\phi_m(r, \omega)$, is obtained from

$$\left[\frac{1}{r}\frac{\partial}{\partial r}\left(r\frac{\partial}{\partial r}\right) - \frac{m^2}{r^2}\right]\; \phi_m(r, \omega) = -4\pi \delta\varrho_m(r, \omega), \tag{S7}$$

where $\delta\varrho_m(r, \omega)$ are the moments of the induced charge density such that

$$\delta\varrho(r, \varphi, \omega) = \frac{1}{2\pi} \sum_{m} \delta\varrho_m(r, \omega)\; e^{im\varphi}. \tag{S8}$$

To address the solution of Eq. (S7), we make use of Green's function

$$G_m(r, r') = \begin{cases} -\frac{1}{2|m|}\left(\frac{r}{r'}\right)^{|m|} & \text{for } r < r' \\ \\ -\frac{1}{2|m|}\left(\frac{r'}{r}\right)^{|m|} & \text{for } r > r' \end{cases}, \tag{S9}$$

which is the solution of the following equation with proper boundary conditions:

$$\left[\frac{1}{r}\frac{\partial}{\partial r}\left(r\frac{\partial}{\partial r}\right)-\frac{m^2}{r^2}\right]\;G_m(r,r')=\frac{1}{r'}\delta(r-r'). \tag{S10}$$

Thus, the radial part of the induced potential can be found from:

$$\begin{aligned}\phi_m(r,\omega)&=-4\pi\int_0^\infty G_m(r,r')\delta\varrho_m(r',\omega)\;r'dr'\\&=2\frac{\pi}{|m|}\left[\int_0^r\left(\frac{r'}{r}\right)^{|m|}\delta\varrho_m(r',\omega)\;r'dr'+\int_r^\infty\left(\frac{r}{r'}\right)^{|m|}\delta\varrho_m(r',\omega)\;r'dr'\right].\end{aligned} \tag{S11}$$

Let us focus on a region well outside the surface of the cylinder such that $r \gg R_c$. Then,

$$\phi_m(r,\omega)=\frac{2\pi}{|m|}\;\int_0^r\left(\frac{r'}{r}\right)^{|m|}\delta\varrho_m(r',\omega)\;r'dr'. \tag{S12}$$

We can rewrite this expression as

$$\phi_m(r,\omega)=2\pi\mathrm{Q}_m(\omega)\left(\frac{R_c}{r}\right)^{|m|}. \tag{S13}$$

Here, $\mathrm{Q}_m(\omega)$ is the multipole moment of the charge density induced in the nanowire per unit length, defined by

$$\begin{aligned}\mathrm{Q}_m(\omega)&=\frac{1}{|m|}\;\int_0^r\left(\frac{r'}{R_c}\right)^{|m|}\;r'dr'\int_{-\pi}^{\pi}\;\delta\varrho(r',\varphi,\omega)\;e^{-im\varphi}\;d\varphi\\&=\frac{1}{|m|}\int_0^r\left(\frac{r'}{R_c}\right)^{|m|}\delta\varrho_m(r',\omega)\;r'dr'.\end{aligned} \tag{S14}$$

Using Eq. (S6) and Eq. (S13) we can write the expression of the induced potential outside the nanowire as

$$V^{\mathrm{ind}}(r,\varphi,\omega)=\sum_m\mathrm{Q}_m(\omega)\left(\frac{R_c}{r}\right)^{|m|}\;e^{im\varphi}. \tag{S15}$$

S3 Induced nonlinear near field

According to the discussion in the main text, for a circularly polarized incident electromagnetic wave, the nonlinear optical response at the nth harmonic of the fundamental frequency

$\omega = n\Omega$ is due to the formation of uniquely the $\mathrm{Q}_n(n\Omega)$ multipolar moment. In this situation, Eq. (S15) results in

$$V^{\mathrm{ind}}(r,\varphi,n\Omega) = \mathrm{Q}_n(n\Omega)\left(\frac{R_c}{r}\right)^n \; e^{in\varphi}. \tag{S16}$$

The induced electric field of the n-th harmonic is given by

$$\mathbf{E}^{\mathrm{ind}}(r,\varphi,n\Omega) = -\nabla V^{\mathrm{ind}}(r,\varphi,n\Omega) = -\hat{\mathbf{e}}_r \frac{\partial V^{\mathrm{ind}}(r,\varphi,n\Omega)}{\partial r} - \hat{\mathbf{e}}_\varphi \frac{1}{r}\frac{\partial V^{\mathrm{ind}}(r,\varphi,n\Omega)}{\partial \varphi}, \tag{S17}$$

which gives the final result

$$\mathbf{E}^{\mathrm{ind}}(r,\varphi,n\Omega) = n\;\frac{R_c^n}{r^{n+1}}\;\mathrm{Q}_n(n\Omega)\;\left[\hat{\mathbf{e}}_r - i\hat{\mathbf{e}}_\varphi\right]\; e^{in\varphi}. \tag{S18}$$

Comparing Eq. (S18) with Eq. (S5), we conclude that the electric near field of the nth harmonic is circularly polarized and rotating clockwise (SAM$= -1$), in contrast to the anti-clockwise (SAM$= +1$) rotation of the incident field.

S4 Linear multipolar polarizabilities

The multipolar polarizability $\alpha_m(\omega)$ is defined as

$$\mathrm{Q}_m(\omega) = \alpha_m(\omega)\;\frac{1}{2\pi} E_m(R_c,\omega), \tag{S19}$$

where $E_m(R_c,\omega)$ is the mth angular harmonic of the radial component of the external field $\mathbf{E}(r,\varphi,\omega)$ evaluated at the nanowire surface

$$E_m(R_c,\omega) = \int_{-\pi}^{\pi} \hat{\mathbf{e}}_r \cdot \mathbf{E}(R_c,\varphi,\omega)\; e^{-im\varphi}\; d\varphi. \tag{S20}$$

Because of rotational symmetry, $\alpha_m(\omega) = \alpha_{-m}(\omega) = \alpha_{|m|}(\omega)$.

To determine $\alpha_m(\omega)$ using TDDFT we follow the electron density dynamics of the system in response to an impulsive potential

$$V(r,\varphi,t) = -\xi\;\delta(t)\;\sum_{m=1}^{m_{\max}} \left(\frac{r}{R_c}\right)^m \cos(m\varphi), \tag{S21}$$

where $\delta(t)$ is the Dirac delta function and $m_{\max}$ is the maximum multipole moment excited in the system (set typically to $m_{\max} \sim 20-40$). The amplitude of the potential, ξ, is sufficiently weak to ensure a linear response as we explicitly check.

Our calculations are performed in 2D cartesian coordinates $\mathbf{r} = (x, y)$ (the problem is translationally invariant with respect to the nanowire axis, chosen as z-axis) . From the time-dependent electron density $n(x, y, t)$ calculated with TDDFT, we obtain the time-dependent multipolar moment $\mathrm{Q}_m(t)$ as

$$\mathrm{Q}_m(t) = \frac{1}{m} \iint \left(\frac{r}{R_c}\right)^m e^{-im\varphi}\ \delta\varrho(x, y, t)\ dx\ dy. \tag{S22}$$

Here, $m = 1, ..., m_{\max}$ and the induced charge density $\delta\varrho(x, y, t)$ is given by

$$\delta\varrho(x, y, t) = -\left[n(x, y, t) - n(x, y, t = 0)\right]. \tag{S23}$$

The frequency-resolved multipolar moment $\mathrm{Q}_m(\omega)$ is obtained from $\mathrm{Q}_m(t)$ using time-to-frequency Fourier transform:

$$\mathrm{Q}_m(\omega) = \int_0^{t_{\max}} dt\ \mathrm{Q}_m(t)\ e^{(i\omega-\eta/2)t}, \tag{S24}$$

where $t_{\max}$ is a finite, albeit large propagation time. An attenuation factor $\eta = 0.15$ eV is introduced to account for dissipation processes such as electron-phonon interaction and inelastic electron-electron scattering that are not included in the adiabatic local density approximation (ALDA) of TDDFT employed here[1,2].

The spectral and angular harmonic decomposition of $V(r, \varphi, t)$ given by Eq. (S21) is

$$V(r, \varphi, \omega) = \frac{1}{2\pi} \sum_{m=-m_{\max}}^{m_{\max}} V_m(r, \omega) e^{im\varphi}, \quad m \neq 0, \tag{S25}$$

where

$$V_m(r, \omega) = -\pi\xi \left(\frac{r}{R_c}\right)^{|m|}. \tag{S26}$$

To derive $V_m(r, \omega)$ we used $\cos(m\varphi) = \frac{1}{2}\left[e^{im\varphi} + e^{-im\varphi}\right]$ and the following convention for

direct and inverse Fourier transforms

$$
\begin{aligned}
\mathcal{F}(\omega) &= \int \mathcal{F}(t) e^{i\omega t} \, dt, \\
\mathcal{F}(t) &= \frac{1}{2\pi} \int \mathcal{F}(\omega) e^{-i\omega t} \, d\omega,
\end{aligned}
\tag{S27}
$$

where $\mathcal{F}$ is a given function.

Considering that

$$
\begin{aligned}
\hat{\mathbf{e}}_r \cdot \mathbf{E}(r, \varphi, \omega) &= -\frac{\partial}{\partial r} V(r, \varphi, \omega) \\
&= \frac{1}{2\pi} \sum_{m=-M}^{M} \pi \xi \, \frac{|m|}{R_c} \left(\frac{r}{R_c} \right)^{|m|-1} e^{im\varphi}, \quad m \neq 0,
\end{aligned}
\tag{S28}
$$

we obtain

$$
\begin{aligned}
\hat{\mathbf{e}}_r \cdot \mathbf{E}(R_c, \varphi, \omega) &= \frac{1}{2\pi} \sum_{m=-M}^{M} \pi \xi \, \frac{|m|}{R_c} e^{im\varphi}, \quad m \neq 0, \\
&= \frac{1}{2\pi} \sum_{m=-M}^{M} E_m(R_c, \omega) e^{im\varphi}, \quad m \neq 0.
\end{aligned}
\tag{S29}
$$

Thus, $\frac{1}{2\pi} E_m(R_c, \omega) = \frac{\xi}{2} \, \frac{|m|}{R_c}$. This expression is substituted in Eq. (S19) together with the frequency-resolved multipolar moment $\mathrm{Q}_m(\omega)$ calculated with TDDFT to determine the linear multipolar polarizability $\alpha_m(\omega)$.

S5 Time-to-frequency Fourier transform of nonlinear quantities

In order to analyze the nonlinear optical response of the nanowire, we first use TDDFT to calculate the time evolution of the physical quantities of interest $\mathcal{F}(t)$. Given $\mathcal{F}(t)$, the frequency-resolved nonlinear quantities $\mathcal{F}(\omega)$ are obtained from a time-to-frequency Fourier transform where we introduce the Gaussian filter given by the envelope of the incident pulse:

$$\mathcal{F}(\omega) = \int dt\ \mathcal{F}(t)\ e^{i\omega t}\ e^{-\left(\frac{t-t_0}{t_p}\right)^2}. \tag{S30}$$

The Gaussian filter in Eq. (S30) partially accounts for dissipation processes beyond ALDA-TDDFT, similarly to the exponential dissipation from Eq. (S24). Most importantly, it enables us to reach convergent spectral response at high-harmonic frequencies[3]. We make use of this approach as the fundamental frequency is off resonance with the dipolar plasmon of the nanowire so that no plasmon ringing and generation of harmonic frequencies is expected when the pulse is over.

S6 Electric field inside a cylindrical nanowire

With the aim of studying the nonlinear polarization vector inside a nanowire, we first derive within the linear-response regime the classical expression of the total electric field inside a metallic cylinder interacting with an incoming monochromatic field which oscillates at frequency Ω. Because of the small diameter of the nanowire, we employ the nonretarded approximation. We search for the total electric field as the sum of the external and induced fields $\mathbf{E}^{\text{tot}}(\mathbf{r},\Omega) = \mathbf{E}(\mathbf{r},\Omega) + \mathbf{E}^{\text{ind}}(\mathbf{r},\Omega)$, where $\mathbf{E}(\mathbf{r},\Omega) = -\nabla V(\mathbf{r},\Omega)$ and $\mathbf{E}^{\text{ind}}(\mathbf{r},\Omega) = -\nabla V^{\text{ind}}(\mathbf{r},\Omega)$. We explicitly split the total potential into the external potential $V(\mathbf{r},\Omega)$ satisfying $\nabla^2 V(\mathbf{r},\Omega) = 0$, and that owing to induced charges, $V^{\text{ind}}(\mathbf{r},\Omega)$. Inside ($r < R_c$) and outside ($r > R_c$) the homogeneous nanowire external charges are absent. Making use of Gauss's law for electric displacement vector, we obtain that $\Delta V^{\text{ind}}(\mathbf{r},\Omega) = 0$.

We can then search for $V^{\text{ind}}(\mathbf{r},\Omega)$ in the form

$$V^{\text{ind}}(r,\varphi,\Omega) = \frac{1}{2\pi}\sum_m V_m^{\text{ind}}(r,\Omega)\ e^{im\varphi}, \tag{S31}$$

where $V_m^{\text{ind}}(r,\Omega)$ fulfills Laplace's equation

$$\left[\frac{1}{r}\frac{d}{dr}r\frac{d}{dr} - \frac{m^2}{r^2}\right] V_m^{\text{ind}}(r,\Omega) = 0. \tag{S32}$$

Imposing $V^{\text{ind}}(\mathbf{r},\Omega)$ to be finite at $r=0$ and at infinity, and considering matching conditions at $r=R_c$, the solution can be written in the form

$$V_m^{\text{ind}}(r,\Omega)=\begin{cases} A_m(\Omega)\ \left(\frac{r}{R_c}\right)^{|m|}, & \text{if } r<R_c \\ A_m(\Omega)\ \left(\frac{R_c}{r}\right)^{|m|}, & \text{if } r>R_c. \end{cases} \tag{S33}$$

The radial component of the induced electric field $E_r^{\text{ind}}(r,\varphi,\Omega)=-\frac{\partial}{\partial r}V^{\text{ind}}(r,\varphi,\Omega)$ is thus given by

$$E_r^{\text{ind}}(r,\varphi,\Omega)=\frac{1}{2\pi}\sum_m E_{r,m}^{\text{ind}}(r,\Omega)\ e^{im\varphi}, \tag{S34}$$

where

$$E_{r,m}^{\text{ind}}(r,\Omega)=\begin{cases} -A_m(\Omega)\ \frac{|m|}{R_c}\left(\frac{r}{R_c}\right)^{|m|-1}, & \text{if } r<R_c \\ A_m(\Omega)\ \frac{|m|}{R_c}\left(\frac{R_c}{r}\right)^{|m|+1}, & \text{if } r>R_c. \end{cases} \tag{S35}$$

For a circularly polarized external field with SAM$=+1$, it follows from Eq. (S5) and $\mathbf{E}(\mathbf{r},\Omega)=-\nabla V(\mathbf{r},\Omega)$ that the external potential is given by

$$V(r,\varphi,\Omega)=-\frac{E_0}{2}re^{i\varphi}, \tag{S36}$$

and the radial component of the external electric field is given by

$$E_r(r,\varphi,\Omega)=\frac{E_0}{2}e^{i\varphi}. \tag{S37}$$

If we express the radial component of the external field in the form

$$E_r(r,\varphi,\Omega)=\frac{1}{2\pi}\sum_m E_{r,m}(r,\Omega)\ e^{im\varphi}, \tag{S38}$$

we obtain

$$\begin{aligned} &E_{r,1}(\Omega)=\pi E_0, \\ &E_{r,m}(\Omega)=0, \quad \text{if} \quad m\neq 1. \end{aligned} \tag{S39}$$

Taking into account the boundary conditions for the radial part of electric displacement

vector $\mathbf{D}$ at R_c we obtain

$$\varepsilon(\Omega)\left(-A_1(\Omega)\,\frac{1}{R_c}+\pi E_0\right)=A_1(\Omega)\,\frac{1}{R_c}+\pi E_0,$$
$$\varepsilon(\Omega)A_m(\Omega)\,\frac{|m|}{R_c}=-A_m(\Omega)\,\frac{|m|}{R_c},\qquad \text{if } m\neq 1. \tag{S40}$$

where $\varepsilon(\Omega)$ is the dielectric function of the nanowire and we assume we are in vacuum. This implies that

$$A_1(\Omega)=\frac{\varepsilon(\Omega)-1}{\varepsilon(\Omega)+1}R_c\pi E_0,$$
$$A_m(\Omega)=0,\qquad \text{if } m\neq 1. \tag{S41}$$

Thus, under circularly polarized illumination with SAM= 1, the total potential inside a cylindrical nanowire can be obtained from equations Eq. (S31), Eq. (S33), Eq. (S36), and Eq. (S41) as

$$V(r,\varphi,\Omega)+V^{\mathrm{ind}}(r,\varphi,\Omega)=-E_0\frac{1}{\varepsilon(\Omega)+1}\;r\;e^{i\varphi},$$
$$=-E_0\frac{1}{\varepsilon(\Omega)+1}\;[x+iy]\,. \tag{S42}$$

Finally, the electric field *inside* the homogeneous cylindrical nanowire $\mathbf{E}^{\mathrm{tot}}=-\nabla\left(V+V^{\mathrm{ind}}\right)$ is given by

$$\mathbf{E}^{\mathrm{tot}}(\mathbf{r},\Omega)=\mathbf{E}^{\mathrm{tot}}(\Omega)=E_0\frac{1}{\varepsilon(\Omega)+1}\left[\hat{\mathbf{e}}_r+i\hat{\mathbf{e}}_\varphi\right]e^{i\varphi},$$
$$=E_0\frac{1}{\varepsilon(\Omega)+1}\left[\hat{\mathbf{e}}_x+i\hat{\mathbf{e}}_y\right]. \tag{S43}$$

Similarly, for a SAM= -1 fundamental field,

$$\mathbf{E}^{\mathrm{tot}}(\mathbf{r},\Omega)=\mathbf{E}^{\mathrm{tot}}(\Omega)=E_0\frac{1}{\varepsilon(\Omega)+1}\left[\hat{\mathbf{e}}_x-i\hat{\mathbf{e}}_y\right]. \tag{S44}$$

Taking into account that a linearly polarized field can be expressed as the superposition of two circularly polarized fields with opposite handedness, for a x-polarized fundamental field

we obtain that

$$\mathbf{E}^{\rm tot}(\mathbf{r},\Omega)=\mathbf{E}^{\rm tot}(\Omega)=E_0\frac{1}{\varepsilon(\Omega)+1}\ \hat{\mathbf{e}}_x. \tag{S45}$$

Thus, inside the nanowire, the total field is homogeneous and its polarization coincides with that of the incident field.

S7 Symmetry-based analysis of the bulk contribution to nonlinear polarization

To derive the selection rules for the bulk nonlinear polarization of a homogeneous free-electron nanowire we will proceed similarly to the derivation of selection rules for the nonlinear multipole moments $\mathrm{Q}_m(n\Omega)$ presented in the main text. We use a general approach[4] based on the symmetry of the system and Neumann's principle for tensors[5,6], and we express the total electric field inside the nanowire $\mathbf{E}^{\rm tot}(\Omega)$ and the nonlinear polarization vector $\mathbf{P}(n\Omega)$ in the basis of the anti-clockwise (SAM = +1)

$$\hat{\mathbf{e}}_{+1}=\frac{1}{\sqrt{2}}\left[\hat{\mathbf{e}}_x+i\ \hat{\mathbf{e}}_y\right]=\frac{1}{\sqrt{2}}\left[\hat{\mathbf{e}}_r+i\hat{\mathbf{e}}_\varphi\right]e^{i\varphi} \tag{S46}$$

and clockwise (SAM = −1) rotating waves

$$\hat{\mathbf{e}}_{-1}=\frac{1}{\sqrt{2}}\left[\hat{\mathbf{e}}_x-i\ \hat{\mathbf{e}}_y\right]=\frac{1}{\sqrt{2}}\left[\hat{\mathbf{e}}_r-i\hat{\mathbf{e}}_\varphi\right]e^{-i\varphi} \tag{S47}$$

as

$$\mathbf{E}^{\rm tot}(\Omega)=\sum_{\mu=\pm1}E_\mu^{\rm tot}(\Omega)\hat{\mathbf{e}}_\mu\ ,\qquad \mathbf{P}(n\Omega)=\sum_{\mu=\pm1}P_\mu(n\Omega)\hat{\mathbf{e}}_\mu\ . \tag{S48}$$

Here, we have taken into account that there is no coordinate dependence of the total field inside the cylinder (see Section S6), and thus the polarization vector is also independent of the spatial coordinates.

For a circularly polarized incoming field with SAM=1, the total field inside the cylinder

maintains the polarization, so that the only nonzero components are $E_1^{\rm tot}(\Omega)$ (be aware that for negative frequency, $E_{-1}^{\rm tot}(-\Omega) = [E_1^{\rm tot}(\Omega)]^*$ holds, where $[Z]^*$ stands for the complex conjugate of complex number Z).

For a fundamental field linearly polarized along the x- axis, the total field inside the cylinder is also x-polarized so that both components are nonzero and $E_1^{\rm tot}(\Omega) = E_{-1}^{\rm tot}(\Omega)$ (for negative frequency, $E_1^{\rm tot}(-\Omega) = E_{-1}^{\rm tot}(-\Omega) = [E_1^{\rm tot}(\Omega)]^*$ holds).

With these definitions, the bulk contribution to the nonlinear polarization corresponding to the n-th harmonic generation with smallest possible nonlinear order in the field can be obtained from

$$P_\nu(n\Omega) = \sum_{\mu_1,\ldots,\mu_n} \mathcal{B}^{(n)}_{\nu;\mu_1\ldots\mu_n} E^{\rm tot}_{\mu_1}(\Omega)\ldots\; E^{\rm tot}_{\mu_n}(\Omega), \tag{S49}$$

where $\nu, \mu_1, \ldots, \mu_n = \pm 1$, and $\mathcal{B}^{(n)}_{\nu;\mu_1\ldots\mu_n} \equiv \mathcal{B}^{(n)}_{\nu;\mu_1\ldots\mu_n}(n\Omega; \underbrace{\Omega, \ldots, \Omega}_{\text{n times}})$ is the nonlinear bulk susceptibility tensor.

Note that since retardation effects can be neglected owing to the small diameter of the nanowire, the total field inside the homogeneous cylinder is position independent as we mentioned before. Therefore, the contribution of nonlinear terms involving spatial derivatives of the field often discussed in the context of the bulk contribution to the second-harmonic generation[7–13] is zero in the present case.

Assume now that we perform an anticlockwise rotation of the x, y-axes by an angle β. In cylindrical coordinates $\varphi = \varphi' + \beta$, where φ' is the azimuthal angle in the rotated coordinate system. Consequently the vector components are transformed as $E_\mu^{\rm tot\,\prime}(\Omega) = e^{+i\mu\beta} E_\mu^{\rm tot}(\Omega)$ and $P'_\mu(n\Omega) = e^{+i\mu\beta} P_\mu(n\Omega)$, and the components of the nonlinear susceptibility tensor are transformed as

$$\left\{\mathcal{B}^{(n)}_{\nu;\mu_1\ldots\mu_n}\right\}' = e^{i(\nu-\mu_1-\ldots-\mu_n)\beta}\; \mathcal{B}^{(n)}_{\nu;\mu_1\ldots\mu_n}. \tag{S50}$$

Neumann's principle[5,6] implies that a rotation by any angle must preserve the form of the

tensor, so that

$$\nu = \mu_1 + ... + \mu_n. \tag{S51}$$

Consider the generation of a harmonic of order $n > 1$. For left-handed circular polarization of the electric field, the only non vanishing component is $E^{\text{tot}}_{+1}(\Omega)$, so that $\mu_1 = ... = \mu_n = 1$. Since $\nu = \pm 1$, Eq. (S51) cannot be satisfied and thus the corresponding tensor element $\mathcal{B}^{(n)}_{\nu\mu_1...\mu_n}$ is zero, so that the nonlinear bulk contribution to the polarization is impossible. This restriction does not hold in the case of linear polarization of the electric field, in which both components $E^{\text{tot}}_{+1}(\Omega)$ and $E^{\text{tot}}_{-1}(\Omega)$ are non-vanishing, and Eq. (S51) can hold for appropriate combinations of $\mu_1, ..., \mu_n$. In this case, the nonlinear polarization in the bulk and harmonic emission into the far field is possible for odd harmonics.

It is worth mentioning that the above proof assumes that the electron gas inside the nanowire is homogeneous. Strictly speaking, for our finite-size system this is not the case because of Friedel oscillations (see Section S10). Notice also that because of the symmetry break at the surface of the nanowire, the surface contribution to the nonlinear response[10,12–16] is possible at any harmonic for both circular and linear polarizations of the external field.

S8 Derivation of the selection rules based on the nonlinear density response formalism

In this section we demonstrate that the nonlinear induced charge density for circularly polarized fundamental field is given by a single Fourier component with order equal to the order of the nonlinearity. More generally, we discuss the symmetry selection rules for the nonlinear charge density induced in a homogeneous metallic nanowire by a circularly or linearly polarized fundamental field. For circular polarization, the potential of the fundamental field is given by

$$V(\mathbf{r}, \Omega) = V_\mu(\Omega) r e^{i\mu\varphi} = -\frac{E_0}{2} r e^{i\mu\varphi}, \tag{S52}$$

where $\mu = +1$ stands for a SAM$= +1$ anti-clockwise rotating field, and $\mu = -1$ stands for a SAM$= -1$ clockwise rotating field (cf. Eq. (S36)). For a linearly, e.g. x-polarized fundamental field the potential is given by

$$V(\mathbf{r}, \Omega) = V_{+1}(\Omega) r e^{i\varphi} + V_{-1}(\Omega) r e^{-i\varphi} = -\frac{E_0}{4} r e^{i\varphi} - \frac{E_0}{4} r e^{-i\varphi} = -\frac{E_0}{2} x. \tag{S53}$$

The n-th harmonic of the induced density can be sought in the general form[17]

$$\begin{aligned} &\delta\varrho(r, \varphi, \Omega_1 + ... + \Omega_n) = \\ &\iint d\mathbf{r}_1 ... \iint d\mathbf{r}_n \; \tilde{\chi}^{(n)}(r, r_1, \varphi - \varphi_1, ..., r_n, \varphi - \varphi_n) \; V(\mathbf{r}_1, \Omega_1) ... \; V(\mathbf{r}_n, \Omega_n), \end{aligned} \tag{S54}$$

where $\tilde{\chi}^{(n)}(\Omega_1 + ... + \Omega_n; r, r_1, \varphi - \varphi_1, ..., r_n, \varphi - \varphi_n) \equiv \tilde{\chi}^{(n)}(r, r_1, \varphi - \varphi_1, ..., r_n, \varphi - \varphi_n)$ is the nonlinear many-body response function. Because of the axial symmetry of the nanowire, this function depends only on the azimuth angle differences $\varphi - \varphi_j$. Introducing the form of the external potential for circular and linear polarizations, Eq. (S54) can be transformed into the following general form

$$\begin{aligned} \delta\varrho(r, \varphi, n\Omega) = \iint d\varphi_1 r_1 dr_1 ... \iint d\varphi_n r_n dr_n \; r_1 ... r_n \; \times \\ \sum_{\mu_1, ..., \mu_n} \tilde{\chi}^{(n)}(r, r_1, \varphi - \varphi_1, ..., r_n, \varphi - \varphi_n) V_{\mu_1}(\Omega) ... \; V_{\mu_n}(\Omega) \; e^{i\mu_1\varphi_1} ... \; e^{i\mu_n\varphi_n}, \end{aligned} \tag{S55}$$

where $\mu_1, ..., \mu_n = \pm 1$ and $V_{\mu_1}(\Omega), ..., V_{\mu_n}(\Omega)$ are defined for circular and linear polarization of the electric field through equations (S52) and (S53) respectively. Note that, in the case of circular polarization with SAM=1, $V_{-1}(\Omega) = 0$ (for SAM=-1, $V_{+1}(\Omega)$=0).

Consider

$$\int_{-\pi}^{\pi} f(\varphi - \varphi_j) \; e^{i\mu\varphi_j} \; d\varphi_j, \tag{S56}$$

where $f(\varphi - \varphi_j)$ is a given function that depends on the azimuth angle difference $\varphi - \varphi_j$.

This integral can be written in the form

$$\int_{-\pi}^{\pi} \frac{1}{2\pi} \sum_m f_m \; e^{im(\varphi-\varphi_j)} \; e^{i\mu\varphi_j} \; d\varphi_j \;\; = \;\; \frac{1}{2\pi} \sum_m f_m \; e^{im\varphi} \; \int_{-\pi}^{\pi} e^{i(\mu-m)\varphi_j} \; d\varphi_j$$
$$= \;\; \sum_m f_m \; e^{im\varphi} \delta_{\mu,m} = f_\mu \; e^{i\mu\varphi}, \tag{S57}$$

where $\delta_{\mu,m}$ is the Kronecker delta and

$$f_m = \int_{-\pi}^{\pi} f(x) \; e^{-imx} \; dx. \tag{S58}$$

Rearranging the terms and integration order, and making use of Eq. (S57), the nonlinear induced density given by Eq. (S55) can be represented in the form

$$\delta\varrho(r, \varphi, n\Omega) = \sum_{\mu_1,\ldots,\mu_n} e^{i\mu_1\varphi} \ldots \; e^{i\mu_n\varphi} \; \chi^{(n)}_{\mu_1,\ldots,\mu_n}(r) \; V_{\mu_1}(\Omega) \ldots \; V_{\mu_n}(\Omega),$$
$$= \sum_{\mu_1,\ldots,\mu_n} e^{i(\mu_1+\mu_2+\ldots+\mu_n)\varphi} \; \chi^{(n)}_{\mu_1,\ldots,\mu_n}(r) \; V_{\mu_1}(\Omega) \ldots \; V_{\mu_n}(\Omega), \tag{S59}$$

where

$$\chi^{(n)}_{\mu_1,\ldots,\mu_n}(r) = \int r_1 dr_1 \ldots \int r_n dr_n \; r_1 \ldots r_n \; \times$$
$$\int_{-\pi}^{\pi} d\vartheta_1 \ldots \int_{-\pi}^{\pi} d\vartheta_n \; \tilde{\chi}^{(n)}(r, r_1, \vartheta_1, \ldots, r_n, \vartheta_n) \; e^{-i\mu_1\vartheta_1} \ldots e^{-i\mu_n\vartheta_n}. \tag{S60}$$

Equation Eq. (S59) can be written in the form

$$\delta\varrho(r, \varphi, n\Omega) = \frac{1}{2\pi} \sum_m \delta\varrho_m(r, n\Omega) \; e^{im\varphi}, \tag{S61}$$

where

$$\delta\varrho_m(r, n\Omega) = \sum_{\substack{\mu_1,\ldots,\mu_n / \\ \mu_1+\mu_2+\ldots+\mu_n=m}} 2\pi\chi^{(n)}_{\mu_1,\ldots,\mu_n}(r) \; V_{\mu_1}(\Omega) \ldots \; V_{\mu_n}(\Omega), \tag{S62}$$

with $\mu_1, \ldots, \mu_n = 1$ for circular polarization with SAM$= +1$, $\mu_1, \ldots, \mu_n = -1$ for circular polarization with SAM$= -1$, and $\mu_1, \ldots, \mu_n = \pm 1$ for linear polarization.

For circular polarization of the fundamental field with SAM$= +1$ (as considered in the

main text), only $V_{+1}(\Omega)$ is non vanishing, so that we obtain

$$\delta\varrho(r,\varphi,n\Omega) = \frac{1}{2\pi}\delta\varrho_n(r,n\Omega)\ e^{in\varphi}, \tag{S63}$$

where

$$\delta\varrho_n(r,n\Omega) = 2\pi\chi^{(n)}_{1,\ldots,1}(r)\ \ [V_1(\Omega)]^n\,. \tag{S64}$$

Thus, the angular dependence of the nonlinear induced charge density at n^{th} harmonic of the fundamental frequency is given by $e^{in\varphi}$, i.e. we retrieve the same constraint as obtained using Neumann's principle for nonlinear multipolar moments.

For a linearly polarized fundamental field both components $V_{\pm 1}(\Omega)$ are present in the potential (see Eq. (S53)). Thus, all terms with $m = \pm|n-2j|$ ($j = 0, 1, ...,$ and $2j < n$) will then contribute to $\delta\varrho(r,\varphi,n\Omega)$ in Eq. (S61). Note that exclusively even (odd) m will be present for even (odd) harmonic order n.

S9 Optical magnetism

As has been recently discussed for a metallic nanoparticle[18], the interaction of circularly polarized light with an axially symmetric plasmonic nanoparticle results in an efficient magnetization of the nanoparticle along its symmetry axis. We observe a similar effect for a nanowire interacting with a Gaussian pulse of left-handed circularly polarized electric field (SAM=1). The permanent magnetic moment $\mathbf{M}_{\text{DC}} = \hat{\mathbf{e}}_z M_z$ excited per unit length in the nanowire is obtained from TDDFT calculations as follows:

$$\begin{aligned} M_z &= \lim_{t\to\infty}\left\{-\frac{1}{2c}\iint \mathbf{r}\times\mathbf{J}(t)\ d^2\mathbf{r}\right\} \\ &= \lim_{t\to\infty}\left\{-\frac{1}{2c}\int dx\int dy\ \ [xJ_y(t) - yJ_x(t)]\right\}, \end{aligned} \tag{S65}$$

where c is the speed of light in vacuum, “minus” sign accounts for the electron charge, and $J_x(t)$ ($J_y(t)$) is the projection of the probability current density $\mathbf{J}(t)$ on x- (y-) axis. $\mathbf{J}(t)$ is

given by

$$\mathbf{J}(t) = \sum_{j\ \in\ \mathrm{occ}} \chi_j\ \mathrm{Im}\left\{[\psi_{\mathrm{j}}(\mathbf{r},\mathrm{t})]^*\ \ \nabla\psi_{\mathrm{j}}(\mathbf{r},\mathrm{t})\right\}, \tag{S66}$$

where the summation runs over occupied Kohn-Sham (KS) orbitals $\psi_j(\mathbf{r}, t)$, Im $\{\mathrm{Z}\}$ stands for the imaginary part of the complex number Z, and the statistical factors $\chi_j = \frac{2\sqrt{2}}{\pi}\sqrt{\mathcal{E}_F - \epsilon_j}$ account for spin degeneracy as well as for the electron motion along the nanowire z-axis. The Fermi energy of the present system is $\mathcal{E}_F = -5.49$ eV with respect to the vacuum level, and ϵ_j stands for the ground-state energies of the KS orbitals.

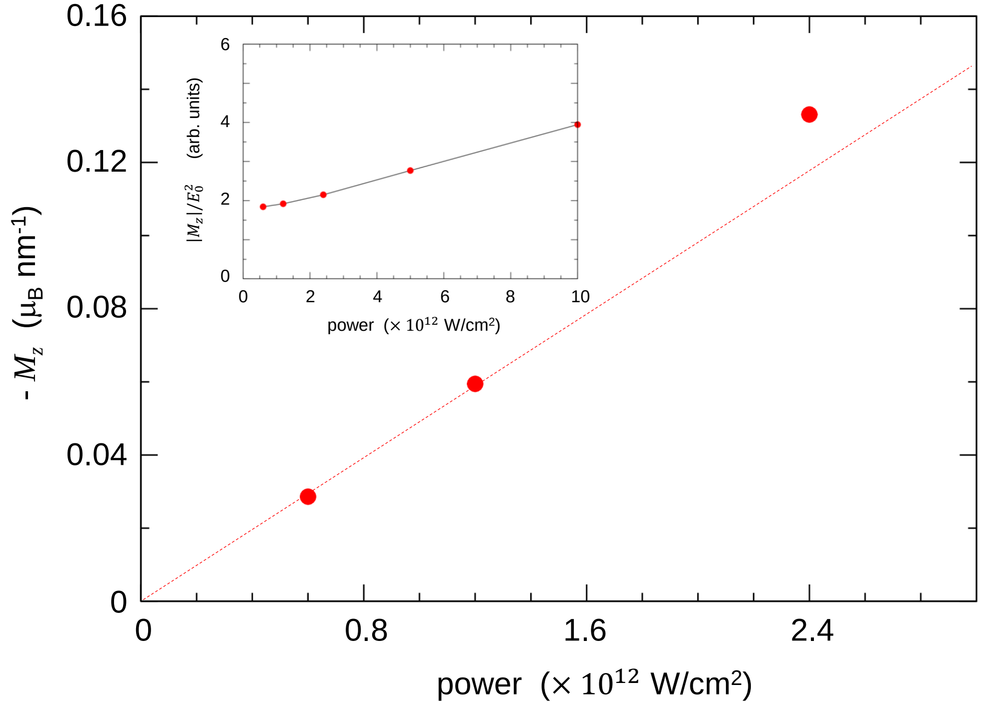

Figure S1: Magnetic moment (in units of Bohr magneton, μ_B, per unit length) induced along the axis of a cylindrical nanowire (z-axis) by a Gaussian pulse with electric field left-handed circularly polarized in the (x, y)-plane. Results are shown as function of the average power of the corresponding optical pulse. The inset presents the normalized quantity $|M_z|/E_0^2$ as a function of the the average power of the corresponding optical pulse.

In Figure S1 we show the calculated permanent magnetic moment of the nanowire induced per unit length by a Gaussian pulse of left-handed circularly polarized electric field (SAM=1) (see Eq. (1) in main text). The results are shown as a function of the average power of the corresponding optical pulse. Observe that $\mathbf{M}_{\mathrm{DC}}$ is oriented in the negative direction of the z-

Table S1: Correspondence between the average power of the circularly polarized electromagnetic pulse and the field amplitude E_0. Further characteristics of the pulse are given in the main text of the paper.

power ($\times 10^{12}$ W/cm^2)	field amplitude, E_0 ($\times 10^{-3}$ a.u.)
0.6	2.6
1.2	3.7
2.4	5.2
5.0	7.5
10.0	10.7

axis. This is consistent with $m \to m+1$ selection rules for the change of the orbital magnetic quantum number upon transitions $j \to j'$ from occupied to unoccupied ground-state KS orbitals with energy difference $\epsilon_{j'} - \epsilon_j = \Omega$. Indeed, if we consider circular polarization (SAM=1) of a monochromatic electric field

$$\begin{aligned} \mathbf{E}(t) &= E_0 \left[\hat{\mathbf{e}}_x \cos(\Omega t) + \hat{\mathbf{e}}_y \sin(\Omega t)\right] \\ &= \frac{E_0}{2}\left[\hat{\mathbf{e}}_x + i\hat{\mathbf{e}}_y\right] e^{-i\Omega t} + \frac{E_0}{2}\left[\hat{\mathbf{e}}_x - i\hat{\mathbf{e}}_y\right] e^{i\Omega t}, \end{aligned} \tag{S67}$$

the potential acting on the nanowire is given within the dipole approximation by

$$\begin{aligned} V(\mathbf{r},t) &= -\frac{E_0}{2}\left[x+iy\right] e^{-i\Omega t} - \frac{E_0}{2}\left[x-iy\right] e^{i\Omega t} \\ &= -\frac{E_0}{2} r \left[e^{i\varphi} e^{-i\Omega t} + e^{-i\varphi} e^{i\Omega t}\right]. \end{aligned} \tag{S68}$$

From perturbation theory and the rotating-wave approximation it follows that transitions are driven by the $e^{-i\Omega t}$ term leading to the above selection rule. As a result of the $m \to m+1$ transitions, upon termination of the pulse the probability current density corresponds to an anti-clockwise rotation of the electrons so that the charge current loop is oriented clockwise.

The order of magnitude of $|M_z|$ shown in Figure S1 agrees with the results obtained in Ref.[18] for an excitation frequency off-resonance with the dipolar plasmon of the nanoparticle (i.e. in similar conditions as here). For low field amplitudes E_0 we obtain that $|M_z| \propto E_0^2$ as expected (for convenience, we give in Table S1 the correspondence between the amplitude E_0 of the electric field used in our calculations and the average power of the corresponding

circularly polarized optical pulse). In contrast, the inset of Figure S1 reveals clear deviations from the square dependence at large E_0. Similarly to Ref. 18, we attribute this effect to the contribution of the photo-emitted electrons. However, note that the main results shown in the paper (i.e., rotation of the induced nonlinear charge density at the fundamental frequency, suppression of far-field emission, and conversion of spin angular momentum of near fields) are not affected by electron emission, as we have explicitly checked.

S10 Friedel oscillations of the electron density

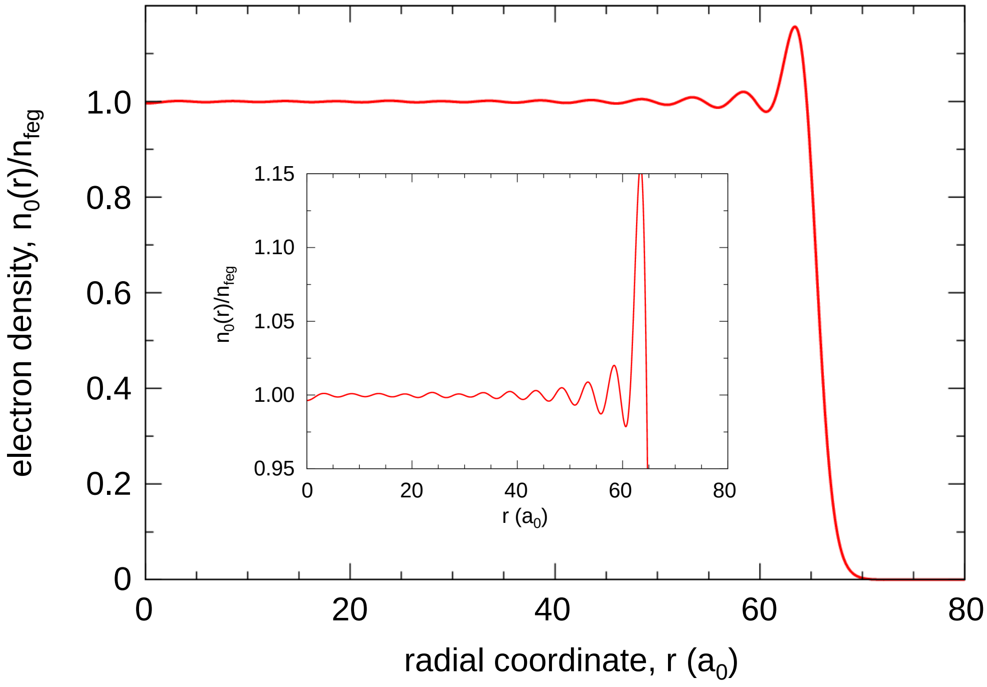

Figure S2: Ground-state electron density $n_0(r)$ of a plasmonic nanowire modelled as a jellium (free-electron-metal) cylinder characterized by the Wigner–Seitz radius of gold $r_s = 3.02$ a_0 ($\mathrm{a}_0 = 0.0529$ nm is the Bohr radius). The nanowire has a radius of $R_c = 66.4$ a_0 (≈ 3.5 nm) and is infinite along the z-axis as sketched in Figure 1 of the main text. We show the normalized density $n_0(r)/n_{\rm feg}$ as function of r, which is measured in units of a_0. Here $n_{\rm feg} = \left[\frac{4\pi}{3} r_s^3\right]^{-1}$ is the bulk electron density of a homogeneous free-electron gas with $r_s = 3.02 a_0$. The inset zooms at the density values close to $n_{\rm feg}$.

In Figure S2 we show the ground-state electron density $n_0(r)$ of a cylindrical nanowire calculated using density functional theory. Here, $\mathbf{r}$ is the 2D position vector given by the

spatial variables (r, φ) (see Figure 1 of main text). Because of symmetry, the ground-state electron density only depends on the radial coordinate r. Finite-size effects (reflection of the electron wave at the nanowire boundary) lead to oscillations in the dependence of $n_0(r)$ on the radial coordinate r (Friedel oscillations). As a consequence, the electron gas within the nanowire is strictly speaking non-homogeneous. This might result in the appearance of a bulk contribution to the nonlinear response in the case of a circularly polarized fundamental field.

S11 Description of movies

We attach to the Supplementary Information 8 movies showing the time evolution of the charge density induced in the nanowire, $\delta\varrho^{(n)}(\mathbf{r}, t) = \mathrm{Re}\left\{\delta\varrho(\mathbf{r}, n\Omega)e^{-in\Omega t}\right\}$, at the fundamental frequency ($n = 1$) and $n = 2, 3, 4$ harmonics for both linear polarization and circular polarization with SAM=1 of the incoming field. The induced charge densities are plotted on a plane transversal to the nanowire axis, and the time evolution is shown over a fundamental period $2\pi/\Omega$. The results correspond to TDDFT calculations for a Gaussian pulse of electric field with fundamental frequency $\Omega = 1.5$ eV and amplitude $E_0 = 1.1 \times 10^{-2}$ a.u., which corresponds to an average power of the circularly polarized pulse of 10^{13} W/cm^2 and twice smaller average power of the linearly polarized pulse. We use a blue-red color scale, where blue (red) corresponds to negative (positive) values of the induced charge density. In each movie, the values are normalized to the maximum absolute value of the corresponding induced charge density over a fundamental period.

References

[S1] Wijewardane, H. O.; Ullrich, C. A. Time-Dependent Kohn-Sham Theory with Memory. *Phys. Rev. Lett.* **2005**, *95*, 086401.

[S2] Vignale, G.; Kohn, W. Current-Dependent Exchange-Correlation Potential for Dynamical Linear Response Theory. *Phys. Rev. Lett.* **1996**, *77*, 2037–2040.

[S3] Babaze, A.; Esteban, R.; Aizpurua, J.; Borisov, A. G. Second-Harmonic Generation from a Quantum Emitter Coupled to a Metallic Nanoantenna. *ACS Photonics* **2020**, *7*, 701–713.

[S4] Konishi, K.; Higuchi, T.; Li, J.; Larsson, J.; Ishii, S.; Kuwata-Gonokami, M. Polarization-Controlled Circular Second-Harmonic Generation from Metal Hole Arrays with Threefold Rotational Symmetry. *Phys. Rev. Lett.* **2014**, *112*, 135502.

[S5] Nye, J. F. *Physical Properties of Crystals, Their Representa- tions by Tensors and Matrices*; Oxford University Press, 1985.

[S6] Alejo-Molina, A.; Hardhienata, H.; Hingerl, K. Simplified bond-hyperpolarizability model of second harmonic generation, group theory, and Neumann's principle. *J. Opt. Soc. Am. B* **2014**, *31*, 526–533.

[S7] Bloembergen, N.; Chang, R. K.; Jha, S. S.; Lee, C. H. Optical Second-Harmonic Generation in Reflection from Media with Inversion Symmetry. *Phys. Rev.* **1968**, *174*, 813–822.

[S8] Rudnick, J.; Stern, E. A. Second-Harmonic Radiation from Metal Surfaces. *Phys. Rev. B* **1971**, *4*, 4274–4290.

[S9] Sipe, J. E.; So, V. C. Y.; Fukui, M.; Stegeman, G. I. Analysis of second-harmonic generation at metal surfaces. *Phys. Rev. B* **1980**, *21*, 4389–4402.

[S10] Bachelier, G.; Butet, J.; Russier-Antoine, I.; Jonin, C.; Benichou, E.; Brevet, P.-F. Origin of optical second-harmonic generation in spherical gold nanoparticles: Local surface and nonlocal bulk contributions. *Phys. Rev. B* **2010**, *82*, 235403.

[S11] Ciracì, C.; Poutrina, E.; Scalora, M.; Smith, D. R. Second-harmonic generation in metallic nanoparticles: Clarification of the role of the surface. *Phys. Rev. B* **2012**, *86*, 115451.

[S12] Butet, J.; Brevet, P.-F.; Martin, O. J. F. Optical Second Harmonic Generation in Plasmonic Nanostructures: From Fundamental Principles to Advanced Applications. *ACS Nano* **2015**, *9*, 10545–10562.

[S13] Panoiu, N. C.; Sha, W. E. I.; Lei, D. Y.; Li, G.-C. Nonlinear optics in plasmonic nanostructures. *Journal of Optics* **2018**, *20*, 083001.

[S14] Lüpke, G.; Bottomley, D. J.; van Driel, H. M. Second- and third-harmonic generation from cubic centrosymmetric crystals with vicinal faces: phenomenological theory and experimenl. *J. Opt. Soc. Am. B* **1994**, *11*, 33–44.

[S15] Krasavin, A. V.; Ginzburg, P.; Zayats, A. V. Free-electron Optical Nonlinearities in Plasmonic Nanostructures: A Review of the Hydrodynamic Description. *Laser & Photonics Reviews* **2018**, *12*, 1700082.

[S16] Timbrell, D.; You, J. W.; Kivshar, Y. S.; Panoiu, N. C. A comparative analysis of surface and bulk contributions to second-harmonic generation in centrosymmetric nanoparticles. *Scientific Reports* **2018**, *8*, 3586.

[S17] Liebsch, A. *Electronic excitations at metal surfaces*; Springer Science & Business Media, 1997.

[S18] Sinha-Roy, R.; Hurst, J.; Manfredi, G.; Hervieux, P.-A. Driving Orbital Magnetism in Metallic Nanoparticles through Circularly Polarized Light: A Real-Time TDDFT Study. *ACS Photonics* **2020**, *7*, 2429–2439.
